# Pregnancy Weight Gain After Gastric Bypass or Sleeve Gastrectomy

**DOI:** 10.1001/jamanetworkopen.2023.46228

**Published:** 2023-12-05

**Authors:** Huiling Xu, Natalie Holowko, Ingmar Näslund, Johan Ottosson, Elizabeth V. Arkema, Martin Neovius, Olof Stephansson, Kari Johansson

**Affiliations:** 1 Clinical Epidemiology Division, Department of Medicine, Solna, Karolinska Institutet, Stockholm, Sweden; 2 Division of Obstetrics, Department of Women’s Health, Karolinska University Hospital, Stockholm, Sweden; 3 Department of Surgery, Faculty of Medicine and Health, Örebro University, Örebro, Sweden

## Abstract

**Importance:**

Pregnancy weight gain may affect the association of bariatric surgery with postsurgery pregnancy outcomes. However, the association of pregnancy weight gain with bariatric surgery is unclear.

**Objective:**

To compare pregnancy weight gain among women with a history of bariatric surgery vs those without and to investigate whether pregnancy weight gain differs by surgical procedure, surgery-to-conception interval, and/or surgery-to-conception weight loss.

**Design, Setting, and Participants:**

This nationwide, population-based matched cohort study was conducted in Sweden from 2014 to 2021. Singleton pregnancies with a history of bariatric surgery were propensity score matched (1:1) to pregnancies without such a history according to early-pregnancy body mass index (BMI), prepregnancy diabetes, prepregnancy hypertension, maternal age, smoking status, education level, height, country of birth, and delivery year. In addition, post–gastric bypass pregnancies were matched to post–sleeve gastrectomy pregnancies using the same matching strategy. Data analysis was performed from November 2022 to May 2023.

**Exposure:**

History of bariatric surgery.

**Main Outcomes and Measures:**

Pregnancy weight gain was standardized by gestational age into early-pregnancy BMI-specific *z *scores.

**Results:**

This study included 12 776 pregnancies, of which 6388 had a history of bariatric surgery and 6388 were matched controls. The mean (SD) age was 31.6 (4.9) years for the surgery group and 31.4 (5.2) for the matched controls, with an early-pregnancy mean (SD) BMI of 29.4 (5.2) in both groups. Across all early-pregnancy BMI strata, women with a history of bariatric surgery had lower pregnancy weight gain than matched controls. The differences in pregnancy weight gain *z *score values between the 2 groups were −0.33 (95% CI, −0.43 to −0.23) for normal weight, −0.33 (95% CI, −0.40 to −0.27) for overweight, −0.21 (95% CI, −0.29 to −0.13) for obese class I, −0.16 (95% CI, −0.29 to −0.03) for obese class II, and −0.08 (95% CI, −0.28 to 0.13) for obese class III. Pregnancy weight gain did not differ by surgical procedure. A shorter surgery-to-conception interval (particularly within 1 year) or lower surgery-to-conception weight loss was associated with lower pregnancy weight gain.

**Conclusions and Relevance:**

In this nationwide matched cohort study, women with a history of bariatric surgery had lower pregnancy weight gain than matched controls with similar early-pregnancy characteristics. Pregnancy weight gain was lower in those with a shorter surgery-to-conception interval or lower surgery-to-conception weight loss, but did not differ by surgical procedure.

## Key Points

**Question:**

Does pregnancy weight gain differ by history of bariatric surgery, surgical procedure, surgery-to-conception interval, and/or surgery-to-conception weight loss?

**Findings:**

This cohort study of 12 776 pregnancies (6388 postsurgery vs 6388 controls) in Sweden found that women with history of bariatric surgery gained less weight during pregnancy than women without such a history but with otherwise similar early-pregnancy characteristics. Pregnancy weight gain did not differ between gastric bypass and sleeve gastrectomy, but pregnancy weight gain was lower in women with a shorter surgery-to-conception interval or lower surgery-to-conception weight loss.

**Meaning:**

Pregnancy weight gain is lower in women with a history of bariatric surgery compared with those without, particularly when the surgery-to-conception interval is shorter.

## Teaser

This cohort study examines pregnancy weight gain among women in Sweden with a history of bariatric surgery vs those without and investigates whether pregnancy weight gain differs by surgical procedure, surgery-to-conception interval, and/or surgery-to-conception weight loss.

## Introduction

The prevalence of obesity, defined as a body mass index (BMI; calculated as weight in kilograms divided by height in meters squared) of 30 or higher, among women of reproductive age has increased globally, posing a risk for adverse maternal and fetal outcomes.^1^ Bariatric surgery is currently the most effective treatment for obesity, with gastric bypass and sleeve gastrectomy being the most common procedures.^2^ Previous studies^3,4,5,6^ have found that women with a history of bariatric surgery, compared with those without such a history but otherwise similar presurgery characteristics, had lower risk of gestational diabetes, preeclampsia, large-for-gestational age (LGA) birth, and cesarean delivery, whereas the risks of small-for-gestational age (SGA) and preterm birth were higher.

Pregnancy weight gain is associated with all these aforementioned outcomes^7,8,9,10,11,12^ and may also influence the association of bariatric surgery with adverse outcomes. However, the association of bariatric surgery with postoperative pregnancy weight gain is not well established. Although most existing studies^13,14,15,16,17,18^ report lower pregnancy weight gain after bariatric surgery, many are limited by a small sample size and varying control groups and surgical procedures and do not consider the impact of prepregnancy BMI or gestational age (eg, a shorter pregnancy time provides less chance of gaining more weight). Furthermore, the impact of time and/or weight loss between the bariatric surgery and conception on postoperative pregnancy weight gain is unclear.^19^ This Swedish nationwide study compares pregnancy weight gain among women with a history of bariatric surgery vs those without and investigates whether pregnancy weight gain after bariatric surgery differs by surgical procedure, surgery-to-conception interval, and/or surgery-to-conception weight loss.

## Methods

### Study Design and Data Source

This nationwide, population-based, matched cohort study used the unique personal identification number assigned to each Swedish resident (at birth or immigration) to link data from the Medical Birth Register and Pregnancy Register to the following Swedish registers: the Scandinavian Obesity Surgery Registry (SOReg), National Patient Register, Prescribed Drug Register, Total Population Register, and Longitudinal Integrated Database for Health Insurance and Labor Market Studies.

This study was approved by the Regional Ethics Committee in Stockholm, Sweden, with amendments. Informed consent was waived because of the study’s register-based design and participant nonidentifiability, in accordance with the Swedish Ethical Review Authority. We followed the Strengthening the Reporting of Observational Studies in Epidemiology (STROBE) reporting guideline for cohort studies.^20^

### Study Population

Of the 906 278 deliveries included in the Swedish Medical Birth Register^21^ and Pregnancy Register^22^ between 2014 and 2021, we excluded multiple births, women without a valid personal identification number, missing gestational age, and bariatric surgical procedures that were not gastric bypass or sleeve gastrectomy. After these exclusions, 861 000 pregnancies remained, of which 10 180 were pregnancies with a history of bariatric surgery. Furthermore, we excluded 313 108 pregnancies with missing early-pregnancy BMI, missing pregnancy weight gain, implausible pregnancy weight gain *z *score values (less than −6 or greater than 6), presurgery BMI less than 30, and an interval of 31 days or longer between the last weight measurement and delivery (eFigure 1 in Supplement 1), of which 3786 pregnancies had a history of bariatric surgery (eTable 1 in Supplement 1).

### Exposure

The primary exposure was a history of bariatric surgery (gastric bypass or sleeve gastrectomy) before conception (the earliest surgery was in 2005). This was obtained from the quality register SOReg, which covers 98% of these procedures in Sweden, as described and validated elsewhere.^23,24^

The history of bariatric surgery before conception was further characterized by the surgery-to-conception interval and surgery-to-conception weight loss. The surgery-to-conception interval was defined as the number of years between surgery and conception, expressed as both a continuous and categorical variable (<1, 1 to <2, 2 to <4 [reference], and ≥4 years). Surgery-to-conception weight loss was defined as the difference in weight (kilograms) measured at the first antenatal visit (median 8.6 gestational weeks) and before surgery.

### Outcome

Pregnancy weight gain was calculated as weight measured at delivery (for 8496 women, weight at delivery was unavailable, so weight at the last antenatal visit within 31 days before delivery was used) minus weight measured at the first antenatal visit. This was then standardized (for gestational age) into pregnancy weight gain *z *scores using previously published Swedish early-pregnancy BMI-specific *z *score charts,^25^ expressed as both a continuous and categorical variable (less than −1, −1 to <1, and ≥1). The *z *scores isolated pregnancy weight gain from gestational duration to overcome the otherwise strong correlation between gestational duration and weight gain.

### Covariates

Early-pregnancy BMI was calculated using measured weight and self-reported height at the first antenatal visit and categorized into the following BMI status ranges: underweight (<18.5), normal weight (18.5-24.9), overweight (25.0-29.9), obese class I (30.0-34.9), obese class II (35.0-39.9), or obese class III (≥40.0). Maternal characteristics were categorized as follows: early pregnancy smoking status (nonsmoker vs 1-9 cigarettes per day or ≥10 cigarettes per day), parity (nulliparous vs parous), mother’s country of birth (Nordic [Sweden, Denmark, Norway, Finland, Iceland] vs non-Nordic), highest achieved education (<10, 10-12, or >12 years), and delivery year (2014-2017 vs 2018-2021). The median height from all registered pregnancies was used for women who gave birth more than once to reduce measurement error and missingness. We defined prepregnancy diabetes using *International Statistical Classification of Diseases and Related Health Problems, Tenth Revision (ICD-10)* codes O24.0-24.3 and E10-14 and Anatomical Therapeutic Chemical (ATC) codes A10A and A10B (women with metformin dispensing for coherent polycystic ovarian syndrome diagnosis were excluded). Prepregnancy hypertension was defined using *ICD-10* codes O10 and I10-I15 and ATC codes C02, C03, C07, C08, and C09. *ICD-10* and ATC data were obtained from the National Patient Register, Medical Birth Register, and Prescribed Drug Register within 12 months before pregnancy.

### Propensity Score Matching

A propensity score was estimated using logistic regression with the categorical covariates (smoking status, country of birth, education level, delivery year, prepregnancy diabetes, and prepregnancy hypertension) and the continuous covariates (maternal age, height, and early-pregnancy BMI). Next, 1:1 matching was performed using the nearest neighbor algorithm without replacement, allowing a maximum caliper width equal to 0.2 of the pooled SD for the logit of the propensity score.^26^ We conducted propensity score matching separately for nulliparous and parous participants within each early-pregnancy BMI category.

### Statistical Analysis

Data analysis was performed from November 2022 to May 2023. Pregnancy characteristics were described using the mean and SD for continuous variables and counts with percentages for categorical variables. Standardized mean differences (SMDs) were examined to evaluate the balance of early-pregnancy characteristics between the surgery group and matched controls, as well as between the sleeve gastrectomy group and matched gastric bypass group. Balance was achieved when the SMD was between −0.10 and 0.10.

#### Propensity Score–Matched Pregnancies

Multivariable linear regression was used to compare pregnancy weight gain *z *scores in pregnancies with and without a history of bariatric surgery, stratified by early-pregnancy BMI weight status. Underweight was excluded owing to the small sample size. Because the analyses were performed at the individual pregnancy level, robust SEs clustered by maternal identification were used to address potential correlations from repeated pregnancies in the same mother. The analyses were also conducted separately for nulliparous and parous women to account further for possible dependence. To reduce residual confounding, we made additional adjustments for imbalanced variables (SMD ≥0.1) from the propensity score matching in each early-pregnancy BMI category (eTable 2 in Supplement 1). Furthermore, we applied the same analytical method to compare pregnancy weight gain after sleeve gastrectomy and gastric bypass, respectively. To facilitate interpretation and comparisons with other studies, *z *score values were converted back to pregnancy weight gain in kilograms at week 40.^25^ In addition, we evaluated adherence to the 2009 pregnancy weight gain guidelines by the US Institute of Medicine (IOM) among the surgery group and matched controls.

#### Surgery-to-Conception Interval and Weight Loss

Among pregnancies with a history of bariatric surgery, we used age-adjusted and parity-adjusted linear regression models with robust sandwich estimators to analyze the associations of surgery-to-conception interval and surgery-to-conception weight loss with pregnancy weight gain *z *scores. To allow for nonlinear associations, the surgery-to-conception interval and surgery-to-conception weight loss were modeled as a restricted cubic spline. The placement and number of knots were evaluated using the Akaike information criteria.^27^ To reduce the influence of outliers, we restricted the surgery-to-conception interval to 10 years or less. In addition, to facilitate interpretation, we treated the surgery-to-conception interval as a categorical variable, used multivariable linear regression to calculate the mean differences in *z* score between groups, and evaluated adherence to the IOM guidelines in each surgery-to-conception interval category. We also assessed the number and percentage of pregnancy outcomes, including SGA, LGA, preterm birth, and acute cesarean delivery, by categories of surgery-to-conception interval and pregnancy weight gain *z *score. Gestational diabetes and preeclampsia were not included because our data set lacked pregnancy weight gain data before the diagnosis of these conditions. Using weight gain after diagnosis could introduce reverse causation issues because weight gain patterns might change after diagnosis. Furthermore, we examined whether there is an interaction between surgery-to-conception interval and weight loss. All statistical analyses were performed with Stata statistical software version 16.0 (StataCorp). Significance was set at 2-sided *P* < .05.

## Results

### Study Population

There were 547 892 pregnancies between 2014 and 2021 that fulfilled our inclusion criteria, of which 6394 had a history of bariatric surgery (905 sleeve gastrectomy and 5489 gastric bypass) (eTable 3 in Supplement 1). To ensure that early-pregnancy characteristics were comparable, we conducted 1:1 propensity score matching, resulting in a total of 12 776 pregnancies: 6388 bariatric surgery pregnancies matched with 6388 controls. Furthermore, within the 6394 pregnancies with a history of bariatric surgery, we performed a 1:1 propensity score matching, yielding 890 gastric bypass matched to 890 sleeve gastrectomy pregnancies. Consequently, 15 pregnancies following sleeve gastrectomy and 4599 pregnancies following gastric bypass were unmatched during the propensity score matching process (eTable 4 in Supplement 1).

### Study Characteristics

The Table presents the baseline characteristics of pregnancies with a history of bariatric surgery vs pregnancies without a history of bariatric surgery, as well as post–gastric bypass pregnancies and post–sleeve gastrectomy pregnancies. The mean (SD) age was 31.6 (4.9) years in the bariatric surgery group and 31.4 (5.2) years in matched controls. The mean (SD) early-pregnancy BMI was 29.4 (5.2) in both groups. The BMI category with the most women at the start of pregnancy was overweight (2606 [40.8%] each in the bariatric surgery and match control groups). The majority of women were nonsmokers (5364 [84.0%] in the bariatric surgery group and 5488 [85.8%] in the matched control group), had 10 to 12 years of education (3672 [57.5%] in the bariatric surgery group and 3809 [59.6%] in the matched control group), and had a previous child (4144 pregnancies [64.9%] each in both groups). The majority were born in a Nordic country (5542 [86.8%] in the bariatric surgery group and 5613 [87.8%] in the matched control group), and most delivered between 2018 and 2021 (3531 [55.3%] in the bariatric surgery group and 3484 [54.5%] in the matched control group). All baseline characteristics were well-balanced for the 2 matching procedures, except for education level within the surgery matching (SMD <0.1) (Table).

**Table.** Maternal Characteristics of Singleton Pregnancies With a History of Bariatric Surgery (Gastric Bypass or Sleeve Gastrectomy) Compared With Matched Controls in Sweden, 2014-2021^a^

Characteristic	Participants, No. (%)					
	Pregnancy with a history of bariatric surgery vs those without			Surgical procedure among pregnancies with a history of bariatric surgery		
	Bariatric surgery (n = 6388)	Matched controls (n = 6388)^b^	SMD	Sleeve gastrectomy (n = 890)	Gastric bypass (n = 890)^b^	SMD
Surgical procedure						
Gastric bypass	5481 (86)	NA	NA	NA	890	NA
Sleeve gastrectomy	904 (14)	NA	NA	890	NA	NA
Surgery-to-conception interval, y						
Mean (SD)	3.8 (2.5)	NA	NA	2.1 (1.4)	4.5 (2.7)	−1.103
<1	763 (11.9)	NA	NA	231 (26.0)	77 (8.7)	0.470
1 to <2	1076 (16.9)			267 (30.0)	112 (12.6)	0.435
2 to <4	1922 (30.1)			290 (32.6)	228 (25.6)	0.154
≥4	2624 (41.1)			102 (11.5)	473 (53.1)	−0.995
Maternal age, mean (SD), y	31.6 (4.9)	31.4 (5.2)	0.016	31.7 (4.8)	31.7 (5.0)	−0.008
Maternal height, mean (SD), cm	167.1 (6.4)	167.0 (6.3)	0.001	166.7 (6.0)	166.8 (6.3)	−0.017
BMI before surgery^c^						
Mean (SD)	42.5 (5.4)	NA	NA	40.3 (5.5)	42.5 (5.1)	−0.409
30.0-34.9	290 (4.5)	NA	NA	129 (14.5)	26 (2.9)	0.419
35.0-39.9	1903 (29.8)			324 (36.4)	289 (32.5)	0.083
40.0-44.9	2376 (37.2)			286 (32.1)	321 (36.1)	−0.083
45.0-49.9	1242 (19.5)			100 (11.2)	179 (20.1)	−0.226
≥50.0	574 (9.0)			51 (5.7)	75 (8.4)	−0.105
Early-pregnancy BMI^c^						
Mean (SD)	29.4 (5.2)	29.4 (5.2)	−0.004	29.4 (5.2)	29.2 (5.0)	−0.004
<18.5	<5 (<0.1)	<5 (<0.1)	0.000	NA	NA	NA
18.5-24.9	1296 (20.3)	1296 (20.3)	0.000	171 (19.2)	171 (19.2)	0.000
25.0-29.9	2606 (40.8)	2606 (40.8)	0.000	368 (41.3)	368 (41.3)	0.000
30.0-34.9	1602 (25.1)	1602 (25.1)	0.000	231 (26.0)	231 (26.0)	0.000
35.0-39.9	626 (9.8)	626 (9.8)	0.000	86 (9.7)	86 (9.7)	0.000
≥40.0	255 (4.0)	255 (4.0)	0.000	34 (3.8)	34 (3.8)	0.000
Smoking status						
Nonsmoker	5364 (84.0)	5488 (85.8)	−0.044	782 (87.9)	772 (86.7)	0.034
1-9 Cigarettes/d	600 (9.4)	546 (8.5)	0.033	60 (6.7)	61 (6.9)	−0.004
≥10 Cigarettes/d	194 (3.0)	156 (2.4)	0.014	19 (2.1)	25 (2.8)	−0.043
Missing	227 (3.6)	204 (3.2)	0.022	29 (3.3)	32 (3.6)	−0.019
Educational level						
<10 y	867 (13.6)	790 (12.4)	0.039	109 (12.2)	127 (14.3)	−0.060
10-12 y	3672 (57.5)	3809 (59.6)	−0.029	447 (50.2)	447 (50.2)	0.000
>12 y	1830 (28.7)	1743 (27.3)	0.023	331 (37.2)	315 (35.4)	0.037
Missing	16 (0.3)	52 (0.8)	−0.11	<5 (0.3)	<5 (0.1)	0.047
Nulliparous	2244 (35.1)	2244 (35.1)	−0.015	342 (38.4)	342 (38.4)	0.044
Nordic born	5542 (86.8)	5613 (87.8)	−0.015	712 (80.0)	729 (81.9)	−0.049
Delivery year						
2014-2017	2854 (44.7)	2910 (45.5)	−0.024	159 (17.9)	142 (16.0)	0.051
2018-2021	3531 (55.3)	3484 (54.5)	0.024	731 (82.1)	748 (84.0)	−0.051
Prepregnancy hypertension^d^	255 (4.0)	214 (3.4)	0.025	38 (4.3)	38 (4.3)	0.000
Prepregnancy diabetes^d^	205 (3.2)	163 (2.6)	0.026	30 (3.4)	35 (3.9)	−0.030
Surgery-to-conception weight change, mean (SD), kg	−36.2 (13.5)	NA	NA	−30.0 (12.2)	−36.1 (13.4)	0.477
Surgery-to-conception BMI change, mean (SD)^c^	−13.1 (4.8)	NA	NA	−11.0 (4.3)	−13.1 (4.7)	0.477

Abbreviations: BMI, body mass index; NA, not applicable; SMD, standardized mean difference.

^a^ The exact numbers were not reported for case numbers less than per cell. Missing data are indicated by NA.

^b^ Propensity score matching was based on maternal age, early-pregnancy BMI, smoking status, education level, height, country of birth, delivery year, prepregnancy diabetes, and prepregnancy hypertension using all available data. Each early-pregnancy BMI categories for nulliparous and parous patients were matched separately.

^c^ BMI is calculated as weight in kilograms divided by height in meters squared.

^d^ Prepregnancy refers to within 12 months before conception.

### Propensity Score–Matched Comparisons

#### Women With vs Without a History of Bariatric Surgery

**Figure 1.**
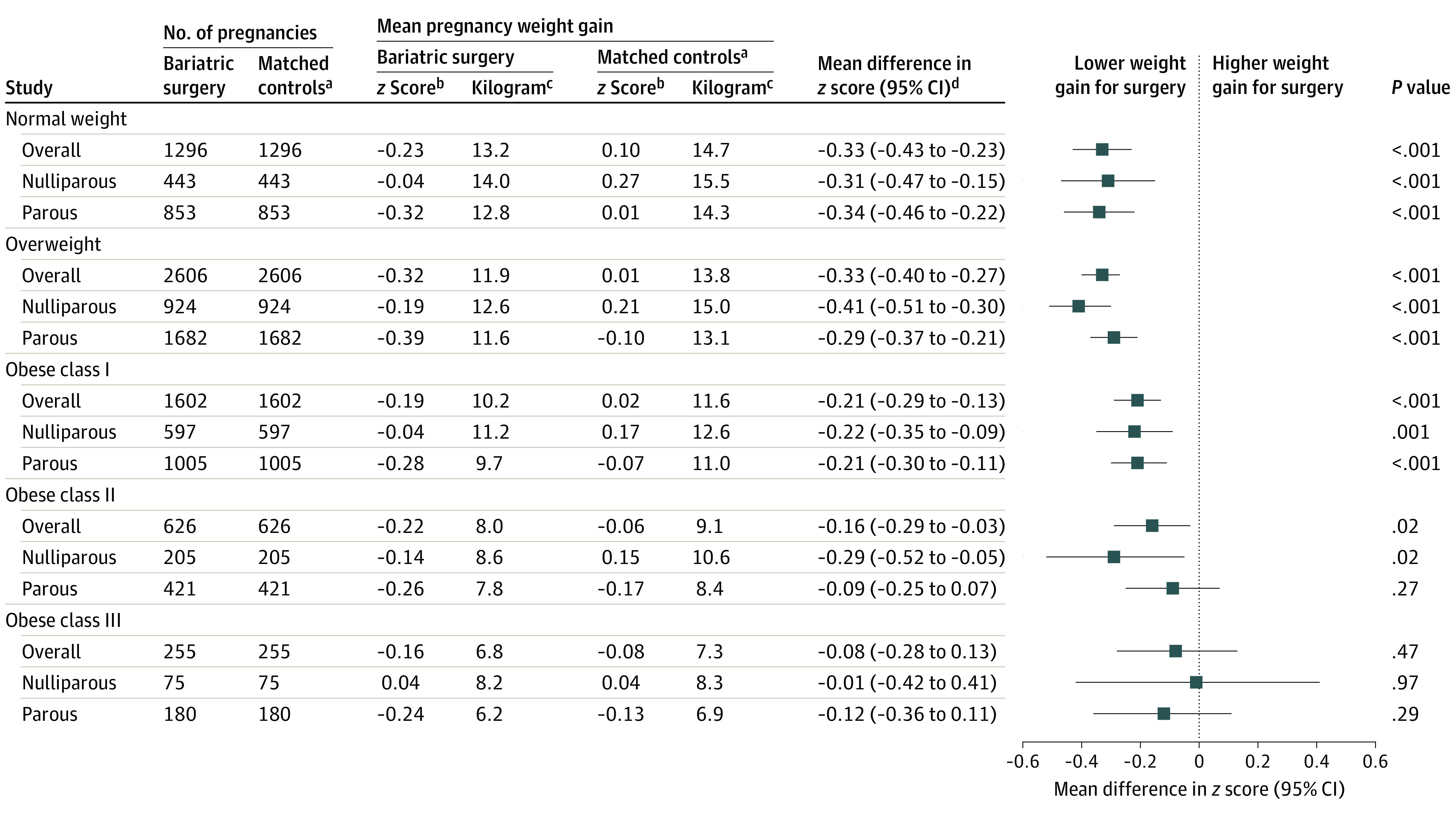
Pregnancy Weight Gain Among Women Who Underwent Bariatric Surgery vs Matched Controls After excluding the underweight group, our analysis included 6385 pregnancies in each group. ^a^Propensity score matching was based on maternal age, early-pregnancy body mass index, smoking status, education level, height, country of birth, delivery year, prepregnancy diabetes, and prepregnancy hypertension using all available data. ^b^The *z *scores refer to the observed mean values, and not estimated means from the regression model. ^c^Refers to corresponding weight gain *z *score in kilogram at 40 weeks. ^d^Mean difference in *z* score was estimated from the multivariable linear regression model with a robust sandwich estimator and also was adjusted for imbalanced variables in each body mass index stratum (specific details are shown in eTable 2 in Supplement 1).

Across all early-pregnancy BMI categories, pregnancy weight gain was lower in women with a history of bariatric surgery, compared with controls matched for early-pregnancy BMI and other factors. The magnitude of difference was largest for women with normal weight or overweight early-pregnancy BMI status, which then decreased stepwise within the obesity subclasses. Among women with a normal weight status, those with a history of bariatric surgery had a pregnancy weight gain *z *score (equivalent to weight in kilograms at week 40) of −0.23 (13.2 kg), and those without had a *z* score of 0.10 (14.7 kg), for an adjusted mean difference of −0.33 (95% CI, −0.43 to −0.23). The respective gains were −0.32 (11.9 kg) and 0.01 (13.8 kg), with an adjusted mean difference of −0.33 (95% CI, −0.40 to −0.27) for women with overweight status. The corresponding gains were −0.19 (10.2 kg) and 0.02 (11.6 kg), with an adjusted mean difference of −0.21 (95% CI, −0.29 to −0.13) for women with obese class I status; −0.22 (8.0 kg) and −0.06 (9.1 kg), with an adjusted mean difference of −0.16 (95% CI, −0.29 to −0.03) for those with obese class II status; and −0.16 (6.8 kg) and −0.08 (7.3 kg), with an adjusted mean difference of −0.08 (95% CI, −0.28 to 0.13) for those with obese class III status (Figure 1). Regardless of early-pregnancy BMI or bariatric surgery history status, nulliparous women gained more weight than parous women.

Pregnancy weight gain below the IOM 2009 recommendations was more common in women with a history of bariatric surgery compared with those without such a history, among women with a normal weight, overweight, and obese class I early-pregnancy BMI status. The largest proportions below the IOM recommendations were found among normal weight women in the surgery group (443 pregnancies [34%] vs 298 pregnancies [23%] in the control group) (eTable 5 in Supplement 1).

#### Sleeve Gastrectomy vs Gastric Bypass

**Figure 2.**
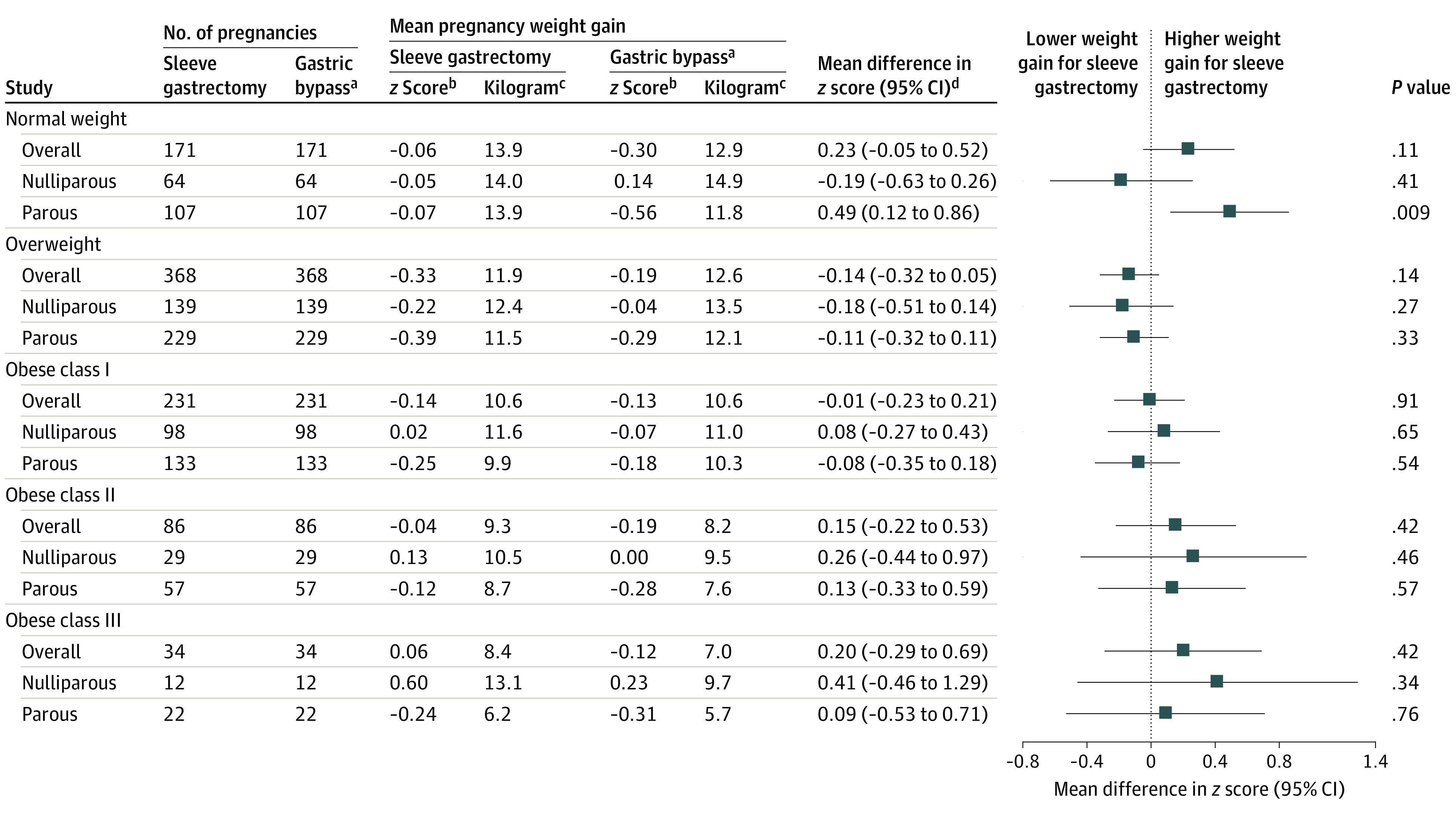
Pregnancy Weight Gain Among Women Who Underwent Sleeve Gastrectomy vs Gastric Bypass ^a^Pregnancies after gastric bypass were matched to those occurring after sleeve gastrectomy using propensity score matching (890 in each group) according to maternal age, early-pregnancy body mass index, smoking status, education level, height, country of birth, delivery year, prepregnancy diabetes, and prepregnancy hypertension, using all available data. ^b^The *z *scores refer to the observed mean values, not estimated means from the regression model. ^c^Refers to corresponding weight gain *z *score in kilograms at 40 weeks. ^d^Mean difference in *z* score was estimated from the multivariable linear regression model with a robust sandwich estimator and also was adjusted for imbalanced variables in each body mass index stratum (specific details are shown in eTable 2 in Supplement 1).

Pregnancy weight gain was similar for women with a history of sleeve gastrectomy and gastric bypass (after matching for early-pregnancy BMI and other factors) and across all early-pregnancy BMI categories. There appeared to be a higher weight gain among women after sleeve gastrectomy with a normal weight status (particularly those who were parous) and obese class II and III weight status; however, the 95% CIs for these estimates were either close to 0 or wide, owing to the small sample (Figure 2).

### Within Bariatric Surgery Comparison

#### Surgery-to-Conception Interval

**Figure 3.**
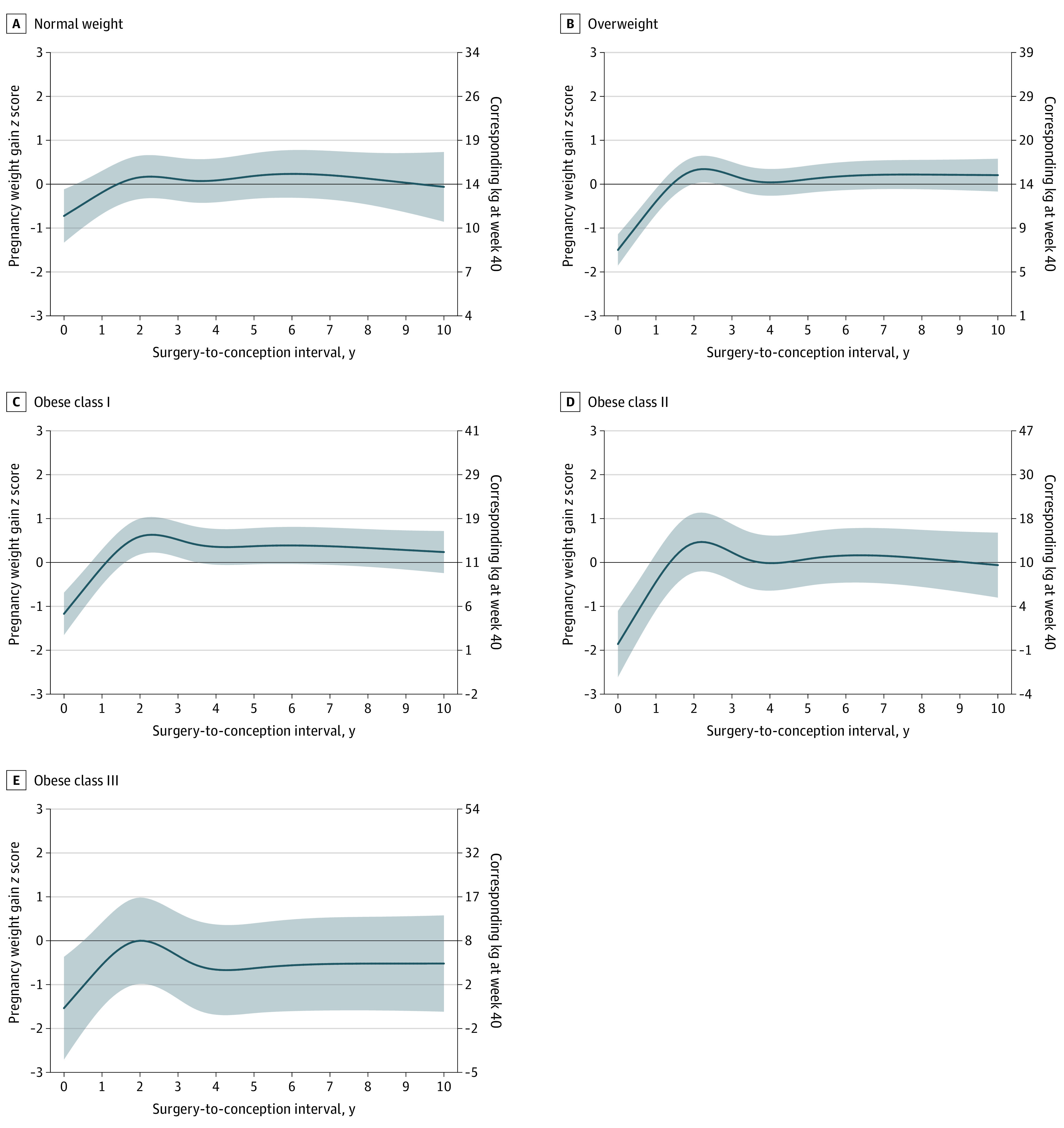
Estimated Means of Pregnancy Weight Gain *z *Scores by Surgery-to-Conception Interval After excluding the underweight group, our analysis included 6391 pregnancies. The solid lines represent the point estimates, and the shaded areas represent the 95% CIs. Estimated means were set at the population average for maternal age and parity. The left y-axis shows pregnancy weight gain *z *scores. The right y-axis represents the corresponding weight gain in kilograms at gestational week 40. The surgery-to-conception interval was modeled using a 5-knot restricted cubic spline.

**Figure 4.**
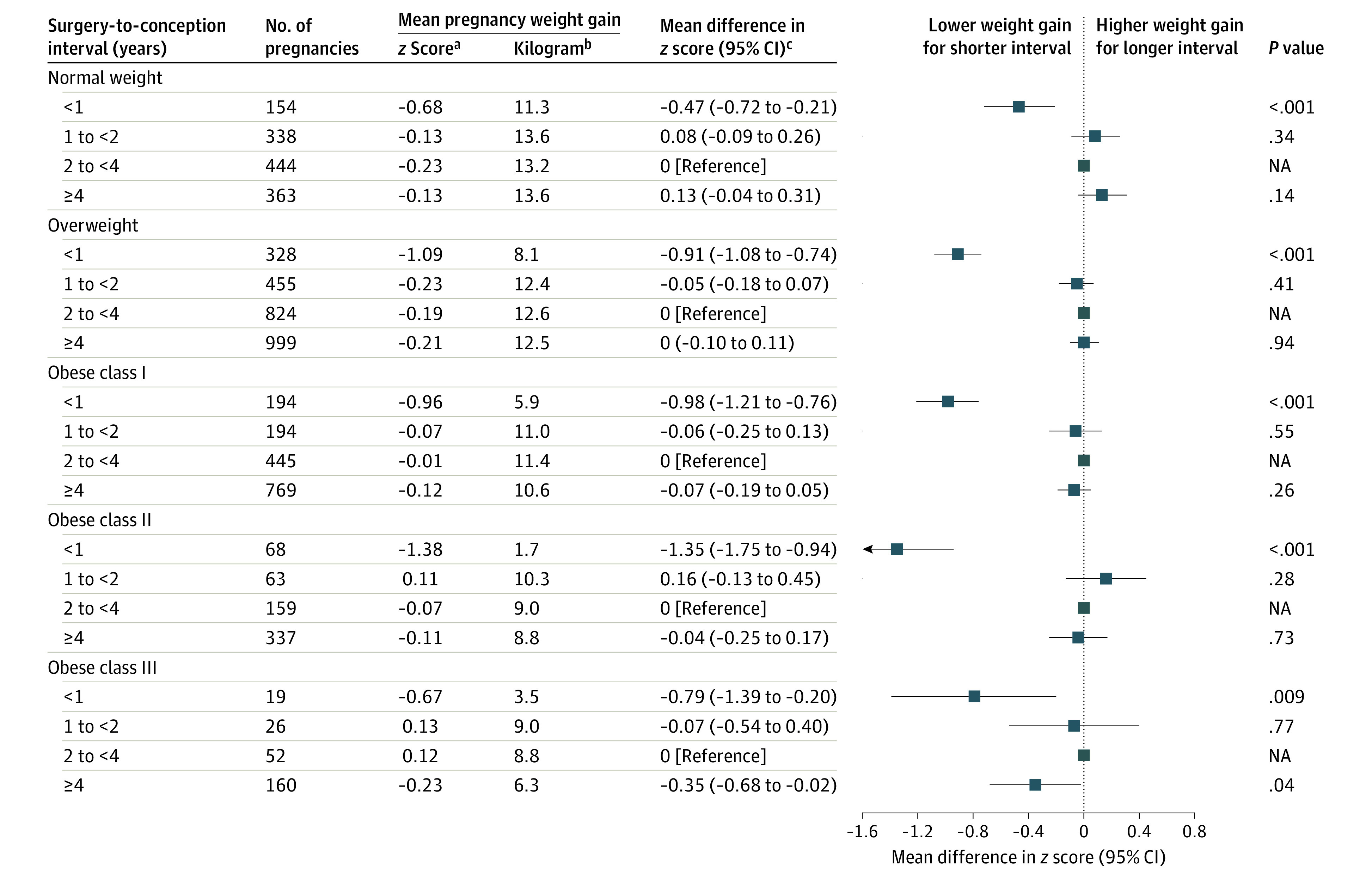
Association of Surgery-to-Conception Interval With Pregnancy Weight Gain Among Pregnancies After Bariatric Surgery After excluding the underweight group, our analysis included 6391 pregnancies. ^a^The *z *scores refer to the observed mean values, not estimated means from the regression model. ^b^Refers to corresponding weight gain *z *score in kilograms at 40 weeks. ^c^Mean difference in *z* score was estimated from the multivariable linear regression model with a robust sandwich estimator and adjustment for maternal age and parity.

Women who conceived within 1 year after bariatric surgery had the lowest pregnancy weight gain. Thereafter, pregnancy weight gain continuously increased until 2 years after surgery, decreasing slightly and remaining fairly stable from 4 to 10 years after surgery (Figure 3; corresponding estimated pregnancy weight gain *z *scores are shown in eTable 6 in Supplement 1). Of note, the estimates after 8 years were unstable owing to the small sample size. Individuals who conceived within 1 year after bariatric surgery vs 2 to less than 4 years after bariatric surgery gained less weight during pregnancy, regardless of early-pregnancy BMI (Figure 4). This association did not differ by surgery type or parity (eFigures 2-5 in Supplement 1). Furthermore, women who became pregnant within 1 year after bariatric surgery had the highest proportion of weight gain below the IOM recommendations.

#### Surgery-to-Conception Weight Loss

For all early-pregnancy BMI categories (except obese class III), there was a positive association between surgery-to-conception weight loss and pregnancy weight gain (eFigure 6 and eTable 7 in Supplement 1). No interaction between surgery-to-conception interval and surgery-to-conception weight loss was found (eTable 8 in Supplement 1).

#### Adverse Pregnancy Outcomes

Among women with a history of bariatric surgery and across all early-pregnancy BMI categories, the highest frequencies of SGA were observed in the lowest pregnancy weight gain category (ie, *z *score category less than −1), whereas the opposite was found for LGA and acute cesarean delivery. For preterm birth, contrasting frequencies were observed across the early-pregnancy BMI categories (eTable 9 in Supplement 1). Furthermore, when considering surgery-to-conception intervals of less than 1 year, we observed similar frequencies of SGA among those with different levels of pregnancy weight gain for women with a normal weight or overweight weight status. However, because of the limited sample size, we could not assess whether other outcomes varied according to pregnancy weight gain *z *score when surgery-to-conception interval less than 1 year.

## Discussion

### Principal Findings

In this cohort study, women with a history of bariatric surgery had lower pregnancy weight gain than those without such a history but otherwise similar early-pregnancy characteristics. Pregnancy weight gain was lower in those with a shorter surgery-to-conception interval or lower surgery-to-conception weight loss but did not differ by surgical procedure.

### Comparison With Other Studies

Previous studies on pregnancy weight gain and bariatric surgery showed inconsistent results. Among 4 studies with a prepregnancy BMI-matched control group, 3 of them^13,16,17^ found lower pregnancy weight gain in postsurgery women, whereas the fourth^28^ reported higher pregnancy weight gain, but the findings in all 4 studies were not statistically significant. We found lower weight gain in pregnancies after bariatric surgery compared with controls matched for early-pregnancy BMI and other characteristics. Notably, previous studies^13,16,17,28^ had small sample size (144 to 307 participants), did not stratify their analysis by prepregnancy or early-pregnancy BMI, and did not consider the effect of gestational age on pregnancy weight gain. To address these limitations, our extensive study was able to attain ample statistical power to stratify the analysis by early-pregnancy BMI. In addition, we used pregnancy weight gain *z *scores to mitigate the association of gestational age with pregnancy weight gain.^29^

The few studies^30,31,32^ that have compared pregnancy weight gain after sleeve gastrectomy and gastric bypass reported no difference, which is consistent with our findings. Unlike previous studies, our study had larger sample size and used propensity score matching to ensure similar characteristics between post–sleeve gastrectomy and post–gastric bypass pregnancies, thus reducing bias due to confounding. In addition, data on the surgical procedure were sourced from the quality register, SOReg, where both a nurse and the performing surgeon enter data, thereby minimizing the risk of exposure misclassification.^23,24^

Existing studies^19,33,34^ on the association between the surgery-to-conception interval and pregnancy weight gain have found, overall, that shorter intervals are associated with lower pregnancy weight gain. However, different comparison intervals were used, which reflects the lack of consensus for the optimal spacing after bariatric surgery (12-18 or 18-24 months until conception, according to current guidelines).^35^ Our study found that women conceiving within 1 year after their surgery gained less weight than women conceiving within 2 to 4 years, regardless of early-pregnancy BMI. Furthermore, we found pregnancy weight gain was lower up until 2 years after surgery and thereafter remaining stable. To our best knowledge, this is the first study investigating the association of surgery-to-conception weight loss with pregnancy weight gain. Furthermore, we found a positive association between surgery-to-conception weight loss and pregnancy weight gain. Greater postsurgery weight loss suggested that women were more likely to start pregnancy with a lower BMI, and thereby have a higher pregnancy weight gain.^36^

### Interpretation

Women with a history of bariatric surgery gained less weight during pregnancy than those with similar BMI entering pregnancy, which may be attributable to physiological effects of altered anatomy and gut hormones, thereby reducing food intake and appetite. In addition, to prevent weight regain after bariatric surgery, women are often advised to avoid excessive pregnancy weight gain.^37^ However, we found that, compared with the controls, a higher proportion of women in the surgery group with a normal, overweight, or obese class I status at the start of pregnancy gained below the pregnancy weight gain guidelines recommended by the IOM. The highest proportion of weight gain below the recommendations was found among women with a normal weight status. Hence, clinical attention to women with history of bariatric surgery and a normal weight status in early pregnancy might be warranted.

### Limitations

Some limitations should be considered when interpreting the results. First, although we included many matching factors in our propensity score modeling and also adjusted for unbalanced factors in each early-pregnancy BMI subgroup, residual confounding may still remain. Second, 3786 pregnancies with a history of bariatric surgery were excluded primarily because of missing pregnancy weight gain data, which may impact the study’s representativeness (eTable 1 in Supplement 1). However, it is unlikely that it would bias our results because there were no major differences between those with vs without pregnancy weight gain data. Third, because of sleeve gastrectomy being a more recent procedure than gastric bypass, pregnancies after sleeve gastrectomy occurred in later years only. Consequently, 15 pregnancies following sleeve gastrectomy and 4599 pregnancies following gastric bypass were unmatched during the propensity score matching process (eTable 4 in Supplement 1); hence, our findings primarily reflect a comparison of post–sleeve gastrectomy and post–gastric bypass pregnancies in recent years. This also led to limited sample sizes, possibly affecting the statistical power. Fourth, our research was not designed to influence the decision-making process for women with obesity contemplating bariatric surgery before pregnancy. Rather, its primary relevance lies in offering clinical insights into weight gain during pregnancy for women who have previously undergone bariatric surgery compared with women without such a history but with otherwise similar early-pregnancy characteristics. Therefore, we used early pregnancy as opposed to presurgery characteristics to conduct the propensity score matching. Fifth, although we have provided an overview of pregnancy outcomes within surgery-to-conception interval and pregnancy weight gain *z *scores, a more in-depth investigation is required to understand the associations among bariatric surgery, pregnancy weight gain, and pregnancy outcomes.

## Conclusions

Women who had undergone bariatric surgery had lower weight gain than those who did not. Pregnancy weight gain was lower in those with a shorter surgery-to conception interval or lower surgery-to conception weight loss, but did not differ by surgical procedure.

## Notes

Supplement 1. **eTable 1.** Maternal Characteristics of Included Versus Excluded Singleton Pregnancies With and Without a History of Bariatric Surgery (Gastric Bypass or Sleeve Gastrectomy) in Sweden Between 2014 and 2021 Before Matching **eTable 2.** Specific Additional Adjustments Based on Early-Pregnancy Weight Status for Women With and Without a History of Bariatric Surgery, and Based on Surgical Procedure **eTable 3.** Maternal Characteristics of Singleton Pregnancies With a History of Bariatric Surgery (Gastric Bypass or Sleeve Gastrectomy), Compared to General Population Pregnancies or by Surgical Procedure, Before Matching in Sweden Between 2014 and 2021 **eTable 4.** Maternal Characteristics of Matched Versus Unmatched Singleton Pregnancies With a History of Bariatric Surgery (Gastric Bypass or Sleeve Gastrectomy) in Sweden Between 2014 and 2021 **eTable 5.** Overview of Adherence to the Institute of Medicine (IOM) Guidelines for Surgery Group, Matched Controls, and Pregnancies Conceived at Different Years After Surgery per Early-Pregnancy BMI Category **eTable 6.** Adjusted Predicted Means of Pregnancy Weight Gain *z* Scores According to Surgery-to-Conception Interval and Stratified by Early-Pregnancy BMI Weight Status **eTable 7.** Adjusted Means of Pregnancy Weight Gain *z* Scores According to Surgery-Conception Weight Change and Stratified by Early-Pregnancy BMI Weight Status **eTable 8.** Means of Pregnancy Weight Gain *z* Scores According to Surgery-to-Conception Interval and Surgery-to-Conception Weight Change, Stratified by Early-Pregnancy BMI Weight Status **eTable 9.** Pregnancy Outcomes by Surgery-to-Conception Interval and Pregnancy Weight Gain *z* Score Categories **eFigure 1.** Inclusion Criteria and Final Sample Size for Pregnancies in Sweden Between 2014-2021 **eFigure 2.** The Association Between Surgery-to-Conception Interval and Pregnancy Weight Gain Among Pregnancies With a History of Gastric Bypass (N=5486) **eFigure 3.** The Association Between Surgery-to-Conception Interval and Pregnancy Weight Gain Among Pregnancies With a History of Sleeve Gastrectomy (N=905) **eFigure 4.** The Association Between Surgery-to-Conception Interval and Pregnancy Weight Gain Among Nulliparous Women With a History of Bariatric Surgery (N=2245) **eFigure 5.** The Association Between Surgery-to-Conception Interval and Pregnancy Weight Gain Among Parous Women With a History of Bariatric Surgery (N=4146) **eFigure 6.** Predicted Means of Pregnancy Weight Gain *z* Scores by Surgery-to-Conception Weight Change (N=6391)

Supplement 2.
Data Sharing Statement
