# Supplementary material for: Pregnancy Weight Gain After Gastric Bypass or Sleeve Gastrectomy
Source: JAMA Netw Open. 2023 Dec 5;6(12):e2346228. doi: 10.1001/jamanetworkopen.2023.46228 (PMC10698625; doi:10.1001/jamanetworkopen.2023.46228)
Supplement: Supplement 1. — eTable 1. Maternal Characteristics of Included Versus Excluded Singleton Pregnancies With and Without a History of Bariatric Surgery (Gastric Bypass or Sleeve Gastrectomy) in Sweden Between 2014 and 2021 Before Matching eTable 2. Specific Additional Adjustments Based on Early-Pregnancy Weight Status for Women With and Without a History of Bariatric Surgery, and Based on Surgical Procedure eTable 3. Maternal Characteristics of Singleton Pregnancies With a History of Bariatric Surgery (Gastric Bypass or Sleeve Gastrectomy), Compared to General Population Pregnancies or by Surgical Procedure, Before Matching in Sweden Between 2014 and 2021 eTable 4. Maternal Characteristics of Matched Versus Unmatched Singleton Pregnancies With a History of Bariatric Surgery (Gastric Bypass or Sleeve Gastrectomy) in Sweden Between 2014 and 2021 eTable 5. Overview of Adherence to the Institute of Medicine (IOM) Guidelines for Surgery Group, Matched Controls, and Pregnancies Conceived at Different Years After Surgery per Early-Pregnancy BMI Category eTable 6. Adjusted Predicted Means of Pregnancy Weight Gain z Scores According to Surgery-to-Conception Interval and Stratified by Early-Pregnancy BMI Weight Status eTable 7. Adjusted Means of Pregnancy Weight Gain z Scores According to Surgery-Conception Weight Change and Stratified by Early-Pregnancy BMI Weight Status eTable 8. Means of Pregnancy Weight Gain z Scores According to Surgery-to-Conception Interval and Surgery-to-Conception Weight Change, Stratified by Early-Pregnancy BMI Weight Status eTable 9. Pregnancy Outcomes by Surgery-to-Conception Interval and Pregnancy Weight Gain z Score Categories eFigure 1. Inclusion Criteria and Final Sample Size for Pregnancies in Sweden Between 2014-2021 eFigure 2. The Association Between Surgery-to-Conception Interval and Pregnancy Weight Gain Among Pregnancies With a History of Gastric Bypass (N=5486) eFigure 3. The Association Between Surgery-to-Conception Interval and Pregnancy Weight Gain Amo [file jamanetwopen-e2346228-s001.pdf]

Supplemental Online Content

Xu H, Holowko N, Näslund I, et al. Pregnancy weight gain after gastric bypass or sleeve gastrectomy. *JAMA Netw Open*. 2023;6(12):e2346228. doi:10.1001/jamanetworkopen.2023.46228

eTable 1. Maternal Characteristics of Included Versus Excluded Singleton Pregnancies With and Without a History of Bariatric Surgery (Gastric Bypass or Sleeve Gastrectomy) in Sweden Between 2014 and 2021 Before Matching

eTable 2. Specific Additional Adjustments Based on Early-Pregnancy Weight Status for Women With and Without a History of Bariatric Surgery, and Based on Surgical Procedure

eTable 3. Maternal Characteristics of Singleton Pregnancies With a History of Bariatric Surgery (Gastric Bypass or Sleeve Gastrectomy), Compared to General Population Pregnancies or by Surgical Procedure, Before Matching in Sweden Between 2014 and 2021

eTable 4. Maternal Characteristics of Matched Versus Unmatched Singleton Pregnancies With a History of Bariatric Surgery (Gastric Bypass or Sleeve Gastrectomy) in Sweden Between 2014 and 2021

eTable 5. Overview of Adherence to the Institute of Medicine (IOM) Guidelines for Surgery Group, Matched Controls, and Pregnancies Conceived at Different Years After Surgery per Early-Pregnancy BMI Category

eTable 6. Adjusted Predicted Means of Pregnancy Weight Gain *z* Scores According to Surgery-to-Conception Interval and Stratified by Early-Pregnancy BMI Weight Status

eTable 7. Adjusted Means of Pregnancy Weight Gain *z* Scores According to Surgery-Conception Weight Change and Stratified by Early-Pregnancy BMI Weight Status

eTable 8. Means of Pregnancy Weight Gain *z* Scores According to Surgery-to-Conception Interval and Surgery-to-Conception Weight Change, Stratified by Early-Pregnancy BMI Weight Status

eTable 9. Pregnancy Outcomes by Surgery-to-Conception Interval and Pregnancy Weight Gain *z* Score Categories

eFigure 1. Inclusion Criteria and Final Sample Size for Pregnancies in Sweden Between 2014-2021

© 2023 Xu H et al. *JAMA Network Open.*

eFigure 2. The Association Between Surgery-to-Conception Interval and Pregnancy Weight Gain Among Pregnancies With a History of Gastric Bypass (N=5486)

eFigure 3. The Association Between Surgery-to-Conception Interval and Pregnancy Weight Gain Among Pregnancies With a History of Sleeve Gastrectomy (N=905)

eFigure 4. The Association Between Surgery-to-Conception Interval and Pregnancy Weight Gain Among Nulliparous Women With a History of Bariatric Surgery (N=2245)

eFigure 5. The Association Between Surgery-to-Conception Interval and Pregnancy Weight Gain Among Parous Women With a History of Bariatric Surgery (N=4146)

eFigure 6. Predicted Means of Pregnancy Weight Gain *z* Scores by Surgery-to-Conception Weight Change (N=6391)

This supplemental material has been provided by the authors to give readers additional information about their work.

eTable 1. Maternal Characteristics of Included Versus Excluded Singleton Pregnancies With and Without a History of Bariatric Surgery (Gastric Bypass or Sleeve Gastrectomy) in Sweden Between 2014 and 2021 Before Matching

	Pregnancy with a history of bariatric surgery (n=10,180)		General population pregnancies (n=850,820)	
	**Included**	**Excluded**	**Included**	**Excluded**
N	6394	3786	541,498	309,322
Surgery-conception-interval, n (%)				
Mean (SD), year	3.8 (2.5)	4.0 (2.8)	NA	NA
<1	764 (12.0)	445 (11.8)		
1 to <2	1077 (16.8)	642 (17.0)		
2 to <4	1924 (30.1)	1082 (28.6)		
≥ 4	2629 (41.1)	1617 (42.7)		
Maternal age, mean (SD), year	31.6 (4.9)	31.9 (5.1)	30.7 (4.9)	30.6 (5.2)
Maternal height, mean (SD), cm	167.1 (6.4)	167.1 (6.5)	166.0 (6.5)	165.9 (6.6)
BMI before surgery, n (%)			NA	NA
Mean (SD), kg/m^2	42.5 (5.4)	42.4 (5.5)		
30.0-34.9	291 (4.6)	164 (4.8)		
35.0-39.9	1908 (29.8)	1008 (29.3)		
40.0-44.9	2377 (37.2)	1306 (38.0)		
45.0-49.9	1243 (19.4)	667 (19.4)		
≥50.0	575 (9.0)	291 (8.5)		
BMI early-pregnancy, n (%)				
Mean (SD), kg/m^2	29.4 (5.2)	29.6 (5.3)	25.0 (4.8)	25.2 (4.9)
<18.5	<5 (0.1)	<5 (0.1)	13,701 (2.5)	6308 (2.4)
18.5-24.9	1299 (20.3)	578 (17.3)	309,126 (57.1)	147,252 (55.5)
25.0-29.9	2606 (40.8)	1415 (42.4)	141,268 (26.1)	71,402 (26.9)
30.0-34.9	1602 (25.0)	847 (25.4)	53,506 (9.9)	28,138 (10.6)
35.0-39.9	627 (9.8)	338 (10.1)	17,577 (3.2)	8905 (3.4)
≥40.0	257 (4.0)	155 (4.6)	6320 (1.2)	3361 (1.3)
Smoking status, n (%)				
Non-smoker	5366 (83.9)	2839 (75.0)	504,738 (93.2)	261,421 (84.5)
1-9 cigarettes/day	604 (9.4)	358 (9.5)	17,306 (3.2)	10,841 (3.5)
≥10 cigarettes/day	196 (3.1)	139 (3.7)	3802 (0.7)	2878 (0.9)
Missing	228 (3.6)	450 (11.9)	15,652 (2.9)	34,182 (11.1)
Educational Level (year), n (%)				
<10 years	871 (13.6)	606 (16.0)	50,292 (9.3)	39,258 (12.7)
10-12 years	3677 (57.5)	2177 (57.5)	186,929 (34.5)	102,140 (33.0)
>12 years	1830 (28.6)	986 (26.0)	296,968 (54.8)	160,872 (52.0)
Missing	16 (0.3)	17 (0.4)	7309 (1.4)	7052 (2.3)
Nulliparous, n (%)	2247 (35.1)	1259 (33.3)	232,977 (43.0)	132,391 (42.8)
Nordic country of birth, n (%)	5550 (86.8)	3275 (86.5)	398,241 (73.5)	214,274 (69.3)
Delivery year				
2014-2017	2858 (44.7)	2138 (56.5)	258,505 (47.7)	182,255 (58.9)
2018-2021	3536 (55.3)	1648 (43.5)	282,993 (52.3)	127,067 (41.1)
Pre-pregnancy hypertension, n (%)[a]	257 (4.0)	167 (4.4)	10,896 (2.0)	6106 (2.0)
Pre-pregnancy diabetes, n (%)[a]	205 (3.2)	152 (4.0)	5788 (1.1)	3910 (1.3)
Surgery-to-conception weight change				
Mean (SD), kg	-36.2 (13.5)	-36.1 (13.5)	NA	NA
Surgery-to-conception BMI change				
Mean (SD), kg/m^2	-13.1 (4.8)	-13.1 (4.8)	NA	NA

Note: The exact numbers were not reported for case number <5 per cell. Missing data was indicated by NA.
[a]Within 12 months before pregnancy.

© 2023 Xu H et al. *JAMA Network Open.*

eTable 2. Specific Additional Adjustments Based on Early-Pregnancy Weight Status for Women With and Without a History of Bariatric Surgery, and Based on Surgical Procedure

	Pregnancies with a history of bariatric surgery vs those without	Pregnancies with a history of sleeve gastrectomy vs gastric bypass
**Normal weight**		
Overall	no additional adjustments	smoking status, education level
Nulliparous	no additional adjustments	education level, height
Parous	education level	smoking status, education level
**Overweight**		
Overall	education level	delivery year
Nulliparous	no additional adjustments	education level, height, country of birth, delivery year
Parous	education level	early-pregnancy BMI, smoking status, delivery year
**Obese I**		
Overall	education level	education level
Nulliparous	education level	pre-pregnancy hypertension
Parous	education level	smoking status, education level
**Obese II**		
Overall	education level	no additional adjustments
Nulliparous	education level, delivery year	age, early-pregnancy BMI, height, smoking status, education level, pre-pregnancy hypertension, pre-pregnancy diabetes
Parous	no additional adjustments	smoking status, education level, height
**Obese III**		
Overall	early-pregnancy BMI, education level, height	age, smoking status, education level, pre-pregnancy diabetes
Nulliparous	early-pregnancy BMI, smoking status, education level, height, pre-pregnancy diabetes, country of birth	age, early-pregnancy BMI, country of birth, delivery year
Parous	early-pregnancy BMI, education level, pre-pregnancy hypertension	age, early-pregnancy BMI, height, smoking status, education level, pre-pregnancy diabetes, country of birth

© 2023 Xu H et al. *JAMA Network Open.*

eTable 3. Maternal Characteristics of Singleton Pregnancies With a History of Bariatric Surgery (Gastric Bypass or Sleeve Gastrectomy), Compared to General Population Pregnancies or by Surgical Procedure, Before Matching in Sweden Between 2014 and 2021

	Pregnancies with a history of bariatric surgery vs those without			Surgery types among within the bariatric surgery group		
	**Bariatric surgery**	**General population pregnancies**	***P* value**	**Sleeve gastrectomy**	**Gastric bypass**	***P* value**
N	6394	541,498	NA	905	5489	NA
Surgery type, n (%)						
GBP	5489 (86)	NA	NA	NA	5489	NA
SG	905 (14)			905	NA	
Surgery-conception-interval, n (%)						
Mean (SD), year	3.8 (2.5)	NA	NA	2.1 (1.4)	4.1 (2.5)	<.001
<1	764 (12.0)	NA	NA	237 (26.2)	527 (9.6)	NA
1 to <2	1077 (16.8)			271 (29.9)	806 (14.7)	
2 to <4	1924 (30.1)			293 (32.4)	1631 (29.7)	
≥ 4	2629 (41.1)			104 (11.5)	2525 (46.0)	
Maternal age, mean (SD), year	31.6 (4.9)	30.7 (4.9)	<.001	31.6 (4.8)	31.6 (4.9)	.90
Maternal height, mean (SD), cm	167.1 (6.4)	166.0 (6.5)	<.001	166.7 (6.1)	167.2 (6.4)	.05
BMI before surgery, n (%)						
Mean (SD), kg/m^2	42.5 (5.4)	NA	<.001	40.3 (5.5)	42.8 (5.3)	<.001
30.0-34.9	291 (4.6)	NA	NA	131 (14.5)	160 (2.9)	<.001
35.0-39.9	1908 (29.8)			329 (36.4)	1579 (28.8)	
40.0-44.9	2377 (37.2)			291 (32.2)	2086 (38.0)	
45.0-49.9	1243 (19.4)			101 (11.2)	1142 (20.8)	
≥50.0	575 (9.0)			53 (5.9)	522 (9.5)	
BMI early-pregnancy, n (%)						
Mean (SD), kg/m^2	29.4 (5.2)	25.0 (4.8)	<.001	29.4 (5.2)	29.4 (5.2)	.90
<18.5	<5 (0.1)	13,701 (2.5)	<.001	<5 (0.0)	<5 (0.1)	.95
18.5-24.9	1299 (20.3)	309,126 (57.1)		176 (19.4)	1123 (20.5)	
25.0-29.9	2606 (40.8)	141,268 (26.1)		370 (40.9)	2236 (40.7)	
30.0-34.9	1602 (25.0)	53,506 (9.9)		234 (25.9)	1,368 (24.9)	
35.0-39.9	627 (9.8)	17,577 (3.2)		89 (9.8)	538 (9.8)	
≥40.0	257 (4.0)	6320 (1.2)		36 (4.0)	221(4.0)	
Smoking status, n (%)						
Non-smoker	5366 (83.9)	504,738 (93.2)	<.001	792 (87.5)	4574 (83.3)	.01
1-9 cigarettes/day	604 (9.4)	17,306 (3.2)		61 (6.7)	543 (9.9)	
≥10 cigarettes/day	196 (3.1)	3802 (0.7)		22 (2.4)	174 (3.2)	
Missing	228 (3.6)	15,652 (2.9)		30 (3.3)	198 (3.6)	
Educational Level, n (%)						
<10 years	871 (13.6)	50,292 (9.3)	<.001	115 (12.7)	756 (13.8)	.001
10-12 years	3677 (57.5)	186,929 (34.5)		449 (49.6)	3228 (58.8)	
>12 years	1830 (28.6)	296,968 (54.8)		338 (37.4)	1492 (27.2)	
Missing	16 (0.3)	7309 (1.4)		<5 (0.3)	13 (0.2)	
Nulliparous, n (%)	2247 (35.1)	232,977 (43.0)	<.001	352 (38.9)	1895 (34.5)	<.001
Nordic country of birth, n (%)	5550 (86.8)	398,241 (73.5)	<.001	714 (78.9)	4836 (88.1)	<.001
Delivery year						
2014-2017	2858 (44.7)	258,505 (47.7)	<.001	160 (17.7)	2698 (49.1)	<.001
2018-2021	3536 (55.3)	282,993 (52.3)		745 (82.3)	2791 (50.9)	
Pre-pregnancy hypertension, n (%)[a]	257 (4.0)	10,896 (2.0)	<.001	40 (4.4)	217 (4.0)	.51
Pre-pregnancy diabetes, n (%)[a]	205 (3.2)	5788 (1.1)	<.001	33 (3.7)	172 (3.1)	.42
Surgery-to-conception weight change						
Mean (SD), kg	-36.2 (13.5)	NA	NA	-30.0 (12.2)	-37.2 (13.5)	<.001
Surgery-to-conception BMI change						
Mean (SD), kg/m^2	-13.1 (4.8)	NA	NA	-11.0 (4.3)	-13.5 (4.8)	<.001

Note: The exact numbers were not reported for case number <5 per cell. Missing data was indicated by NA.
[a]Within 12 months before pregnancy

© 2023 Xu H et al. *JAMA Network Open.*

eTable 4. Maternal Characteristics of Matched Versus Unmatched Singleton Pregnancies With a History of Bariatric Surgery (Gastric Bypass or Sleeve Gastrectomy) in Sweden Between 2014 and 2021

	Pregnancy with a history of bariatric surgery		Surgery type among pregnancies with a history of bariatric surgery			
	Matched	Unmatched	Matched Sleeve gastrectomy	Unmatched Sleeve gastrectomy	Matched Gastric bypass	Unmatched Gastric bypass
N	6388	6	890	15	890	4599
Surgery type, n (%)						
Gastric bypass	5481 (86)	5 (83)	NA	NA	890	4599
Sleeve gastrectomy	904 (14)	<5 (17)	890	15	NA	NA
Surgery-conception-interval, n (%)						
Mean (SD), year	3.8 (2.5)	5.6 (2.2)	2.1 (1.4)	1.9 (1.6)	4.5 (2.7)	4.0 (2.5)
<1	763 (11.9)	<5 (33.3)	231 (26.0)	6 (40.0)	77 (8.7)	449 (9.8)
1 to <2	1076 (16.9)	<5 (66.7)	267 (30.0)	<5 (26.7)	112 (12.6)	693 (15.1)
2 to <4	1922 (30.1)	NA	290 (32.6)	<5 (20.0)	228 (25.6)	1403 (30.5)
≥4	2624 (41.1)	NA	102 (11.5)	<5 (13.3)	473 (53.1)	2051 (44.6)
Maternal age, mean (SD), year	31.6 (4.9)	39.4 (6.2)	31.7 (4.8)	29.9 (5.9)	31.7 (5.0)	31.6 (4.9)
Maternal height, mean (SD), cm	167.1 (6.4)	172.9 (4.4)	166.7 (6.0)	164.7 (8.6)	166.8 (6.3)	167.2 (6.4)
BMI before surgery, n (%)						
Mean (SD), kg/m^2	42.5 (5.4)	44.1 (11.0)	40.3 (5.5)	41.1 (6.8)	42.5 (5.1)	42.9 (5.4)
30.0 – 34.9	290 (4.5)	<5 (49.9)	129 (14.5)	<5 (13.3)	26 (2.9)	133 (2.9)
35.0 – 39.9	1903 (29.8)	<5 (16.7)	324 (36.4)	<5 (33.3)	289 (32.5)	1288 (28.0)
40 0 – 44.9	2376 (37.2)	<5 (16.7)	286 (32.2)	<5 (33.3)	321 (36.1)	1765 (38.4)
45.0 – 49.9	1242 (19.5)	<5 (16.7)	100 (11.2)	<5 (6.8)	179 (20.1)	963 (21.0)
≥50.0	574 (9.0)	NA	51 (5.7)	<5 (13.3)	75 (8.4)	447 (9.7)
BMI early-pregnancy, n (%)						
Mean (SD), kg/m^2	29.4 (5.2)	32.1 (8.2)	29.4 (5.2)	30.5 (8.6)	29.2 (5.0)	29.4 (5.2)
<18.5	<5 (0.0)	NA	NA	NA	NA	NA
18.5 - 24.9	1296 (20.3)	<5 (50.0)	171 (19.2)	5 (33.3)	171 (19.2)	952 (20.7)
25.0 - 29.9	2606 (40.8)	<5 (16.7)	368 (41.3)	<5 (13.4)	368 (41.3)	1868 (40.6)
30.0 - 34.9	1602 (25.1)	<5 (33.3)	231 (26.0)	<5 (20.0)	231 (26.0)	1137 (24.7)
35.0 - 39.9	626 (9.8)	NA	86 (9.7)	<5 (20.0)	86 (9.7)	452 (9.8)
≥40.0	255 (4.0)	NA	34 (3.8)	<5 (13.3)	34 (3.8)	187 (4.1)
Smoking status, n (%)						
Non-smoker	5364 (84.0)	<5 (33.3)	782 (87.9)	10 (66.7)	772 (86.7)	3802 (82.7)
1-9 cigarettes/day	600 (9.4)	<5 (16.7)	60 (6.7)	<5 (6.7)	61 (6.9)	479 (10.4)
≥10 cigarettes/day	194 (3.0)	<5 (33.3)	19 (2.1)	<5 (19.9)	25 (2.8)	149 (3.2)
Missing	227 (3.6)	<5 (16.7)	29 (3.3)	<5 (6.7)	32 (3.6)	166 (3.6)
Educational level, n (%)						
<10 years	867 (13.6)	<5 (16.7)	109 (12.2)	6 (40.0)	127 (14.3)	626 (13.6)
10-12 years	3672 (57.5)	5 (83.3)	447 (50.2)	<5 (13.3)	447 (50.2)	2781 (60.5)
>12 years	1830 (28.7)	NA	331 (37.2)	7 (46.7)	315 (35.4)	1177 (25.6)
Missing	16 (0.3)	NA	<5 (0.3)	<5 (0.0)	<5 (0.1)	12 (0.3)
Nulliparous, n (%)	2244 (35.1)	<5 (16.7)	342 (38.4)	10 (66.7)	342 (38.4)	1551 (33.7)
Nordic born, n (%)	5542 (86.8)	5 (83.3)	712 (80.0)	<5 (13.3)	729 (81.9)	4104 (89.3)
Delivery year						
2014-2017	2854 (44.7)	<5 (66.7)	159 (17.9)	<5 (6.7)	142 (16.0)	2556 (55.6)
2018-2021	3531 (55.3)	<5 (33.3)	731 (82.1)	14 (93.3)	748 (84.0)	2040 (44.4)
Pre-pregnancy hypertension, n (%)[a]	255 (4.0)	<5 (33.3)	38 (4.3)	<5 (13.3)	38 (4.3)	179 (3.9)
Pre-pregnancy diabetes, n (%)[a]	205 (3.2)	NA	30 (3.4)	<5 (20.0)	35 (3.9)	137 (3.0)
Surgery-to-conception weight change						
Mean (SD), kg	-36.2 (13.5)	-36.0 (26.2)	-30.0 (12.2)	-27.7 (8.6)	-36.1 (13.4)	-37.4 (13.5)
Surgery-to-conception BMI change						
Mean (SD), kg/m^2	-13.1 (4.8)	-12.0 (8.9)	-11.0 (4.3)	1.9 (1.6)	-13.1 (4.7)	4.0 (2.5)

Note: The exact numbers were not reported for case number <5 per cell. Missing data was indicated by NA.
[a]Within 12 months before pregnancy

eTable 5. Overview of Adherence to the Institute of Medicine (IOM) Guidelines for Surgery Group, Matched Controls, and Pregnancies Conceived at Different Years After Surgery per Early-Pregnancy BMI Category

	IOM Recommendation, kg	Corresponding z-scores at 40 weeks	n (%)					
			Bariatric surgery (n=6385)	Matched controls (n=6385)	Surgery-to-conception interval, year (n=6391)			
					<1	1 to <2	2 to <4	≥4
**Normal weight**								
Below	<11.5	<-0.63	443 (34)	298 (23)	74 (48)	105 (31)	147 (33)	118 (33)
Adequate	11.5 to 16	-0.63 to 0.37	427 (33)	463 (36)	45 (29)	105 (31)	151 (34)	126 (35)
Above	>16	>0.37	426 (33)	535 (41)	35 (23)	128 (38)	146 (33)	119 (33)
**Overweight**								
Below	<11.5	<-1.3	472 (18)	298 (11)	135 (41)	73 (16)	111 (14)	153 (15)
Adequate	7 to 11.5	-1.3 to -0.4	655 (25)	547 (21)	89 (27)	105 (23)	214 (26)	247 (25)
Above	>11.5	>-0.4	1479 (57)	1761 (68)	104 (32)	277 (61)	499 (61)	599 (60)
**Obese class I**								
Below	<5	<-1.1	308 (19)	211 (13)	88 (45)	29 (15)	66 (15)	125 (16)
Adequate	5 to 9	-1.1 to -0.4	283 (18)	275 (17)	26 (13)	33 (17)	82 (18)	142 (19)
Above	>9	>-0.4	1011 (63)	1116 (70)	80 (41)	132 (68)	297 (67)	502 (65)
**Obese class II**								
Below	<5	<-1.1	109 (17)	94 (15)	33 (49)	7 (11)	21 (13)	48 (14)
Adequate	5 to 9	-1.1 to -0.4	112 (18)	120 (19)	14 (21)	10 (16)	27 (17)	61 (18)
Above	>9	>-0.4	405 (65)	412 (66)	21 (31)	46 (73)	111 (70)	228 (68)
**Obese class III**								
Below	<5	<-1.1	41 (16)	37 (15)	6 (32)	<5 (12)	6 (12)	26 (16)
Adequate	5 to 9	-1.1 to -0.4	44 (17)	50 (20)	<5 (21)	7 (27)	7 (14)	27 (17)
Above	>9	>-0.4	170 (67)	168 (66)	9 (47)	16 (62)	39 (75)	107 (67)

Note: The exact numbers were not reported for case number <5 per cell. Missing data was indicated by NA.

© 2023 Xu H et al. *JAMA Network Open.*

eTable 6. Adjusted Predicted Means of Pregnancy Weight Gain *z* Scores According to Surgery-to-Conception Interval and Stratified by Early-Pregnancy BMI Weight Status

Early-pregnancy BMI	Surgery-to-conception interval, year[a]	Adjusted mean pregnancy weight gain z-score (95%CI)[b]
**Normal weight**		
	0.5	-0.45 (-0.99, 0.08)
	1	-0.19 (-0.68, 0.30)
	1.5	0.03 (-0.46, 0.52)
	2	0.16 (-0.34, 0.65)
	2.5	0.16 (-0.32, 0.65)
	3	0.11 (-0.38, 0.60)
	4	0.09 (-0.41, 0.58)
	5	0.19 (-0.33, 0.70)
	10	-0.06 (-0.86, 0.73)
**Overweight**		
	0.5	-0.94 (-1.25, -0.63)
	1	-0.40 (-0.69, -0.10)
	1.5	0.06 (-0.24, 0.36)
	2	0.31 (0.01, 0.62)
	2.5	0.33 (0.02, 0.63)
	3	0.20 (-0.10, 0.51)
	4	0.04 (-0.26, 0.35)
	5	0.11 (-0.20, 0.42)
	10	0.21 (-0.17, 0.58)
**Obese class I**		
	0.5	-0.63 (-1.06, -0.21)
	1	-0.11 (-0.51, 0.29)
	1.5	0.33 (-0.07, 0.74)
	2	0.59 (0.18, 1.00)
	2.5	0.62 (0.22, 1.02)
	3	0.52 (0.12, 0.92)
	4	0.36 (-0.05, 0.76)
	5	0.37 (-0.04, 0.78)
	10	0.24 (-0.24, 0.72)
**Obese class II**		
	0.5	-1.14 (-1.82, -0.47)
	1	-0.44 (-1.09, 0.20)
	1.5	0.13 (-0.52, 0.79)
	2	0.44 (-0.23, 1.11)
	2.5	0.42 (-0.24, 1.08)
	3	0.23 (-0.41, 0.87)
	4	-0.02 (-0.64, 0.61)
	5	0.08 (-0.53, 0.69)
	10	-0.06 (-0.80, 0.68)
**Obese class III**		
	0.5	-1.04 (-2.07, -0.01)
	1	-0.55 (-1.52, 0.41)
	1.5	-0.16 (-1.14, 0.81)
	2	-0.00 (-0.98, 0.98)
	2.5	-0.11 (-1.08, 0.87)
	3	-0.34 (-1.32, 0.64)
	4	-0.66 (-1.69, 0.37)
	5	-0.63 (-1.65, 0.40)
	10	-0.52 (-1.62, 0.58)

[a]The interval was chosen based on the turning point in our predicted curve. For example, the peak is around 2-3 years, and the relative evening out from 4 -5 years to 10 years. Additionally, as the various guidelines suggested postponing 12-24 months to get pregnant, we added predicted values for 0.5, 1, 1,5, and 2 years.
[b]Predicted mean pregnancy weight gain z-scores were adjusted for maternal age and parity.

© 2023 Xu H et al. *JAMA Network Open.*

eTable 7. Adjusted Means of Pregnancy Weight Gain *z* Scores According to Surgery-Conception Weight Change and Stratified by Early-Pregnancy BMI Weight Status

Early-pregnancy BMI	Amount of weight change, kg[a]	Adjusted mean pregnancy z-score (95% CI)[b]
**Normal weight**		
	-126	0.73 (-0.19, 1.65)
	-100	0.46 (-0.25, 1.16)
	-80	0.25 (-0.32, 0.82)
	-60	0.04 (-0.45, 0.52)
	-40	-0.17 (-0.64, 0.31)
	-20	-0.31 (-0.90, 0.29)
	0	-0.42 (-1.43, 0.59)
	23	-0.55 (-2.13, 1.03)
**Overweight**		
	-126	0.86 (0.20, 1.53)
	-100	0.46 (-0.04, 0.96)
	-80	0.14 (-0.24, 0.53)
	-60	-0.17 (-0.48, 0.14)
	-40	-0.47 (-0.77, -0.17)
	-20	-0.68 (-1.02, -0.34)
	0	-0.85 (-1.34, -0.37)
	23	-1.06 (-1.76, - 0.35)
**Obese class I**		
	-126	0.26 (-0.57, 1.09)
	-100	0.18 (-0.45, 0.80)
	-80	0.11 (-0.38, 0.61)
	-60	0.04 (-0.37, 0.46)
	-40	-0.05 (-0.46, 0.37)
	-20	-0.38 (-0.81, 0.05)
	0	-0.81 (-1.32, -0.31)
	23	-1.30 (-1.96, -0.65)
**Obese class II**		
	-126	1.18 (-0.09, 2.46)
	-100	0.73 (-0.24, 1.71)
	-80	0.39 (-0.40, 1.18)
	-60	0.04 (-0.64, 0.73)
	-40	-0.28 (-0.97, 0.41)
	-20	-0.32 (-0.98, 0.34)
	0	-0.27 (-0.93, 0.39)
	23	-0.21 (-0.97, 0.55)
**Obese class III**		
	-126	-0.38 (-2.36, 1.61)
	-100	-0.38 (-1.96, 1.20)
	-80	-0.39 (-1.70, 0.92)
	-60	-0.39 (-1.49, 0.70)
	-40	-0.41 (-1.39, 0.58)
	-20	-0.53 (-1.50, 0.43)
	0	-0.70 (-1.75, 0.35)
	23	-0.88 (-2.14, 0.37)

[a]We selected the weight change interval based on the turning point in our predicted curve, which was around -40 to -20 kg. As -120 and 20 kg were not present in our data, we used -126 and 23 kg instead.
[b]Predicted mean pregnancy weight gain z-scores were adjusted for maternal age and parity.

© 2023 Xu H et al. *JAMA Network Open.*

eTable 8. Means of Pregnancy Weight Gain *z* Scores According to Surgery-to-Conception Interval and Surgery-to-Conception Weight Change, Stratified by Early-Pregnancy BMI Weight Status

	Surgery-to-conception interval, year	GWG z-scores (kg), means (SD) Surgery-to-conception weight change[a]			
		<-45kg	-45 to <-36 kg	-36 to <-28 kg	≥-28 kg
**Normal Weight**					
	<1	-0.58 (11.7)	-0.73 (11.1)	-0.67 (11.3)	-0.75 (11.0)
	1 to <2	-0.01 (14.2)	-0.18 (13.4)	-0.37 (12.6)	-0.01 (14.1)
	2 to <4	-0.12 (13.7)	-0.30 (12.9)	-0.27 (13.0)	-0.37 (12.5)
	≥ 4	0.04 (14.4)	-0.31 (12.8)	-0.12 (13.7)	-0.38 (12.5)
**Overweight**					
	<1	-0.75 (9.7)	-1.08 (8.2)	-1.23 (7.5)	-1.13 (5.6)
	1 to <2	-0.11 (13.1)	-0.18 (12.7)	-0.32 (11.9)	-0.39 (9.2)
	2 to <4	-0.02 (13.6)	-0.14 (12.9)	-0.21 (12.5)	-0.46 (10.1)
	≥ 4	-0.09 (13.2)	-0.16 (12.8)	-0.27 (12.2)	-0.34 (9.5)
**Obese class I**					
	<1	-1.07 (5.4)	-0.84 (6.5)	-0.94 (6.0)	-1.02 (5.6)
	1 to <2	0.13 (12.3)	0.28 (13.3)	-0.17 (10.4)	-0.35 (9.2)
	2 to <4	0.25 (13.1)	0.11 (12.2)	-0.05 (11.1)	-0.22 (10.1)
	≥ 4	0.12 (12.2)	0.02 (11.6)	-0.03 (11.2)	-0.32 (9.5)
**Obese class II**					
	<1	-0.80 (4.5)	-1.38 (1.7)	-1.45 (1.4)	-1.49 (1.2)
	1 to <2	0.03 (9.7)	0.20 (11.0)	-0.16 (8.4)	0.28 (11.6)
	2 to <4	0.22 (11.1)	-0.35 (7.2)	0.23 (11.2)	-0.20 (8.1)
	≥ 4	0.29 (11.7)	-0.13 (8.6)	-0.05 (9.2)	-0.17 (8.3)
**Obese class III**					
	<1	0.62 (13.3)	0.21 (9.6)	-1.51 (-0.6)	-0.82 (2.7)
	1 to <2	-0.34 (5.5)	0.79 (15.0)	-0.03 (7.7)	0.04 (8.2)
	2 to <4	-0.25 (6.1)	0.09 (8.7)	0.67 (13.8)	0.03 (8.2)
	≥ 4	0.12 (8.9)	-0.28 (5.9)	-0.29 (5.8)	-0.25 (6.1)

[a]Surgery-to-conception weight change was categorized into <-45kg, -45 to -36kg, -36 to -28kg, and ≥ -28kg according to the 25, 50, 75 percentiles.

© 2023 Xu H et al. *JAMA Network Open.*

eTable 9. Pregnancy Outcomes by Surgery-to-Conception Interval and Pregnancy Weight Gain *z* Score Categories

Outcomes (n/N [%])	Surgery-to-conception interval, year	Pregnancy weight gain z-score categories, n (%)		
		<-1	-1 to <1	≥1
**SGA (n=769/6394 [12.2%])**				
**Normal weight (n=214)**	**Overall**	**71 (23.4)**	**129 (16.1)**	**14 (7.3)**
	<1	11 (50.0)	10 (45.5)	<5 (4.5)
	1 to <2	26 (39.4)	35 (53.0)	5 (7.6)
	2 to <4	16 (22.9)	51 (72.9)	<5 (4.3)
	≥4	18 (32.1)	33 (58.9)	5 (8.9)
**Overweight (n=301)**	**Overall**	**97 (14.7)**	**183 (11.0)**	**21 (7.4)**
	<1	20 (47.6)	19 (45.2)	<5 (7.1)
	1 to <2	12 (21.1)	43 (75.4)	<5 (3.5)
	2 to <4	27 (30.7)	49 (55.7)	12 (13.6)
	≥4	38 (33.3)	72 (63.2)	<5 (3.5)
**Obese class I (n=157)**	**Overall**	**50 (15.0)**	**92 (8.4)**	**15 (8.3)**
	<1	15 (68.2)	<5 (18.2)	<5 (13.6)
	1 to <2	<5 (13.3)	10 (66.7)	<5 (20.0)
	2 to <4	10 (21.3)	34 (72.3)	<5 (6.4)
	≥4	23 (31.5)	44 (60.3)	6 (8.2)
**Obese class II (n=65)**	**Overall**	**24 (20.9)**	**35 (8.1)**	**6 (7.6)**
	<1	9 (81.8)	<5 (18.2)	<5 (0.0)
	1 to <2	<5 (37.5)	<5 (50.0)	<5 (12.5)
	2 to <4	5 (29.4)	10 (58.8)	<5 (11.8)
	≥4	7 (24.1)	19 (65.5)	<5 (10.3)
**Obese class III (n=32)**	**Overall**	**8 (17.4)**	**21 (11.4)**	**<5 (11.1)**
	<1	<5 (50.0)	<5 (50.0)	<5 (0.0)
	1 to <2	<5 (40.0)	<5 (60.0)	<5 (0.0)
	2 to <4	<5 (0.0)	6 (100.0)	<5 (0.0)
	≥4	<5 (23.5)	10 (58.8)	<5 (17.6)
**LGA (n=536/6394 [8.5%])**				
**Normal weight (n=47)**	**Overall**	**5 (1.7)**	**26 (3.2)**	**16 (8.3)**
	<1	<5 (0.0)	<5 (100.0)	<5 (0.0)
	1 to <2	<5 (25.0)	<5 (50.0)	<5 (25.0)
	2 to <4	<5 (14.3)	11 (52.4)	7 (33.3)
	≥4	<5 (5.6)	9 (50.0)	8 (44.4)
**Overweight (n=179)**	**Overall**	**25 (3.8)**	**105 (6.3)**	**49 (17.3)**
	<1	<5 (5.6)	14 (77.8)	<5 (16.7)
	1 to <2	<5 (12.5)	17 (70.8)	<5 (16.7)
	2 to <4	10 (17.5)	33 (57.9)	14 (24.6)
	≥4	11 (13.8)	41 (51.2)	28 (35.0)
**Obese class I (n=184)**	**Overall**	**24 (7.2)**	**124 (11.4)**	**36 (20.0)**
	<1	<5 (9.1)	6 (54.5)	<5 (36.4)
	1 to <2	<5 (23.1)	8 (61.5)	<5 (15.4)
	2 to <4	<5 (6.2)	33 (68.8)	12 (25.0)
	≥4	17 (15.2)	77 (68.8)	18 (16.1)
**Obese class II (n=79)**	**Overall**	**8 (7.0)**	**61 (14.1)**	**10 (12.7)**
	<1	<5 (0.0)	<5 (100.0)	<5 (0.0)
	1 to <2	<5 (0.0)	7 (77.8)	<5 (22.2)
	2 to <4	<5 (4.8)	19 (90.5)	<5 (4.8)
	≥4	7 (15.2)	32 (69.6)	7 (15.2)
**Obese class III (n=47)**	**Overall**	**6 (13.0)**	**32 (17.4)**	**9 (33.3)**
	<1	<5 (100.0)	<5 (0.0)	<5 (0.0)
	1 to <2	<5 (0.0)	<5 (50.0)	<5 (50.0)
	2 to <4	<5 (20.0)	6 (60.0)	<5 (20.0)
	≥4	<5 (9.4)	24 (75.0)	5 (15.6)

© 2023 Xu H et al. *JAMA Network Open.*

Outcomes (n/N [%])	Surgery-to-conception interval, year	Pregnancy weight gain z-score categories, n (%)		
		<-1	-1 to <1	≥1
**Preterm (n=418/6394 [6.5%])**				
**Normal weight (n=96)**	**Overall**	**33 (10.9)**	**52 (6.5)**	**11 (5.7)**
	<1	8 (57.1)	6 (42.9)	<5 (0.0)
	1 to <2	7 (36.8)	11 (57.9)	<5 (5.3)
	2 to <4	9 (26.5)	21 (61.8)	<5 (11.8)
	≥4	9 (31.0)	14 (48.3)	6 (20.7)
**Overweight (n=168)**	**Overall**	**45 (6.8)**	**99 (6.0)**	**24 (8.5)**
	<1	11 (57.9)	8 (42.1)	<5 (0.0)
	1 to <2	8 (30.8)	15 (57.7)	<5 (11.5)
	2 to <4	13 (21.3)	37 (60.7)	11 (18.0)
	≥4	13 (21.0)	39 (62.9)	10 (16.1)
**Obese class I (n=105)**	**Overall**	**25 (7.5)**	**63 (5.8)**	**17 (9.4)**
	<1	8 (80.0)	<5 (20.0)	<5 (0.0)
	1 to <2	<5 (7.1)	12 (85.7)	<5 (7.1)
	2 to <4	6 (14.6)	28 (68.3)	7 (17.1)
	≥4	10 (25.0)	21 (52.5)	9 (22.5)
**Obese class II (n=33)**	**Overall**	**5 (4.3)**	**20 (4.6)**	**8 (10.1)**
	<1	<5 (0.0)	<5 (100.0)	<5 (0.0)
	1 to <2	<5 (0.0)	<5 (0.0)	<5 (100.0)
	2 to <4	<5 (11.1)	6 (66.7)	<5 (22.2)
	≥4	<5 (19.0)	13 (61.9)	<5 (19.0)
**Obese class III (n=16)**	**Overall**	**<5 (4.3)**	**11 (6.0)**	**<5 (11.1)**
	<1	<5 (0.0)	<5 (100.0)	<5 (0.0)
	1 to <2	<5 (50.0)	<5 (50.0)	<5 (0.0)
	2 to <4	<5 (8.3)	8 (66.7)	<5 (25.0)
	≥4	NA	NA	NA
**Acute C-section (n=450/6394 [7.0%])**				
**Normal weight (n=62)**	**Overall**	**14 (4.6)**	**34 (4.2)**	**14 (7.3)**
	<1	<5 (20.0)	<5 (60.0)	<5 (20.0)
	1 to <2	<5 (30.8)	5 (38.5)	<5 (30.8)
	2 to <4	5 (19.2)	18 (69.2)	<5 (11.5)
	≥4	<5 (22.2)	8 (44.4)	6 (33.3)
**Overweight (n=160)**	**Overall**	**36 (5.4)**	**99 (6.0)**	**25 (8.8)**
	<1	10 (52.6)	8 (42.1)	<5 (5.3)
	1 to <2	<5 (6.5)	20 (64.5)	9 (29.0)
	2 to <4	11 (22.0)	30 (60.0)	9 (18.0)
	≥4	13 (21.7)	41 (68.3)	6 (10.0)
**Obese class I (n=133)**	**Overall**	**21 (6.3)**	**91 (8.4)**	**21 (11.7)**
	<1	5 (38.5)	6 (46.2)	<5 (15.4)
	1 to <2	<5 (13.3)	10 (66.7)	<5 (20.0)
	2 to <4	<5 (11.4)	26 (74.3)	5 (14.3)
	≥4	10 (14.3)	49 (70.0)	11 (15.7)
**Obese class II (n=65)**	**Overall**	**7 (6.1)**	**51 (11.8)**	**7 (8.9)**
	<1	<5 (20.0)	8 (80.0)	<5 (0.0)
	1 to <2	<5 (0.0)	7 (70.0)	<5 (30.0)
	2 to <4	<5 (12.5)	12 (75.0)	<5 (12.5)
	≥4	<5 (10.3)	24 (82.8)	<5 (6.9)
**Obese class III (n=30)**	**Overall**	<5 **(6.5)**	**21 (11.4)**	**6 (22.2)**
	<1	<5 (20.0)	<5 (60.0)	<5 (20.0)
	1 to <2	<5 (0.0)	<5 (50.0)	<5 (50.0)
	2 to <4	<5 (0.0)	7 (100.0)	<5 (0.0)
	≥4	<5 (14.3)	9 (64.3)	<5 (21.4)

The exact numbers were not reported for case number <5 per cell. Missing data was indicated by NA.

© 2023 Xu H et al. *JAMA Network Open.*

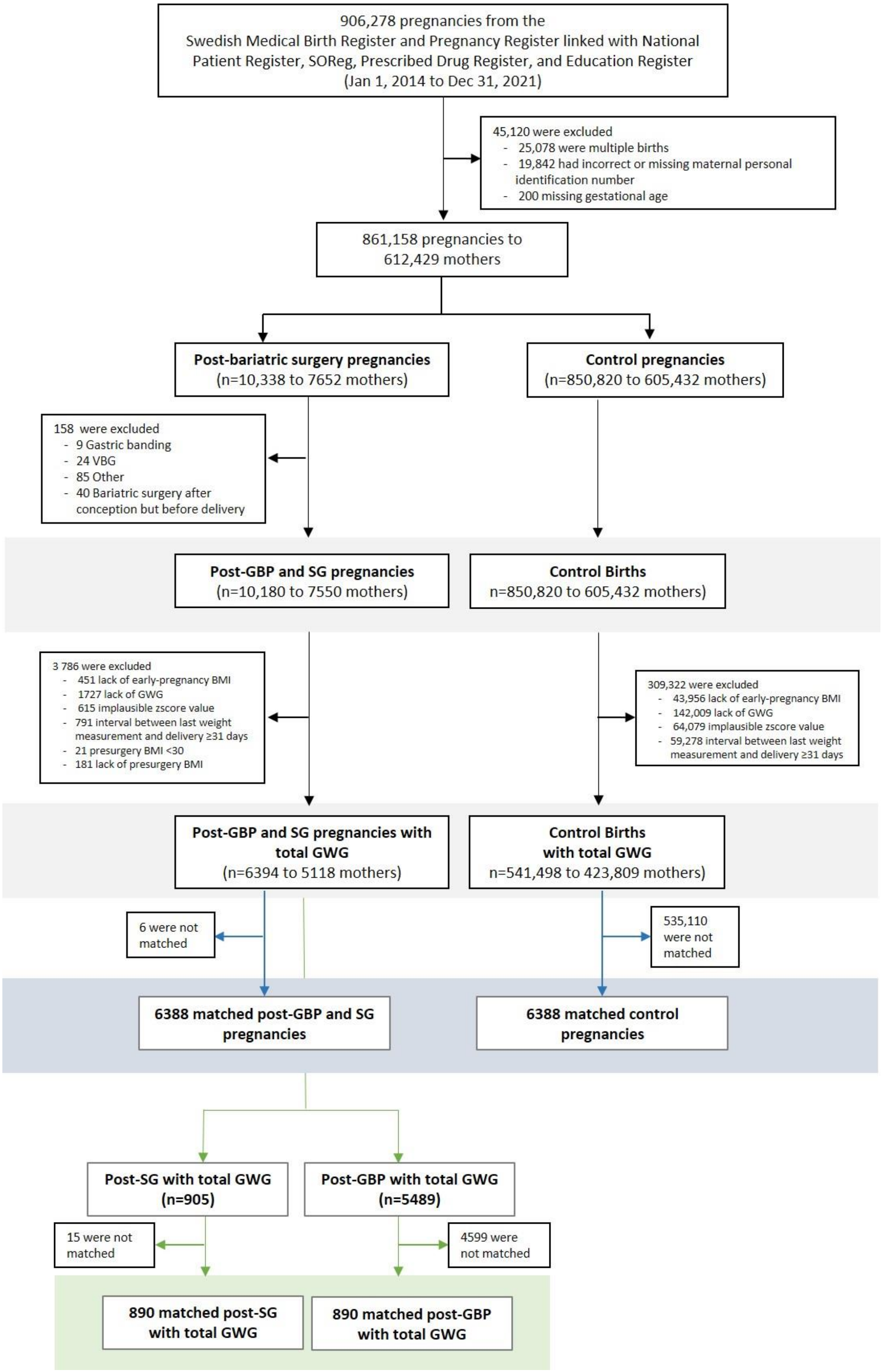

eFigure 1. Inclusion Criteria and Final Sample Size for Pregnancies in Sweden Between 2014-2021

© 2023 Xu H et al. *JAMA Network Open.*

Surgery-to-conception interval (years)	No of pregnancies	Mean pregnancy weight gain Z-score[a]	Kilogram[b]	Mean difference in z score (95% CI)[c]	P value
**Normal weight**					
<1	109	-0.72	11.1	-0.45 (-0.75, -0.16)	.003
1 to <2	269	-0.19	13.4	0.09 (-0.10, 0.28)	.35
2 to <4	391	-0.29	12.9	Ref	NA
≥4	354	-0.13	13.6	0.20 (0.01, 0.38)	.04
**Overweight**					
<1	220	-1.12	8.0	-0.93 (-1.13, -0.73)	<.001
1 to <2	353	-0.26	12.2	-0.06 (-0.21, 0.08)	.39
2 to <4	707	-0.22	12.4	Ref	NA
≥4	956	-0.22	12.4	0.02 (-0.09, 0.12)	.75
**Obese class I**					
<1	132	-1.13	5.1	-1.16 (-1.42, -0.90)	<.001
1 to <2	124	-0.25	9.9	-0.24 (-0.48, 0.00)	.05
2 to <4	370	-0.01	11.4	Ref	NA
≥4	742	-0.11	10.7	-0.05 (-0.18, 0.08)	.42
**Obese class II**					
<1	51	-1.56	0.9	-1.52 (-2.00, -1.05)	<.001
1 to <2	43	-0.05	9.1	0.01 (-0.37, 0.38)	.98
2 to <4	123	-0.08	9.0	Ref	NA
≥4	321	-0.11	8.7	-0.03 (-0.25, 0.19)	.81
**Obese class III**					
<1	14	-0.72	3.2	-0.90 (-1.66, -0.15)	.02
1 to <2	16	-0.23	6.2	-0.46 (-0.96, 0.04)	.07
2 to <4	40	0.17	9.3	Ref	NA
≥4	151	-0.22	6.3	-0.40 (-0.76, -0.03)	.03

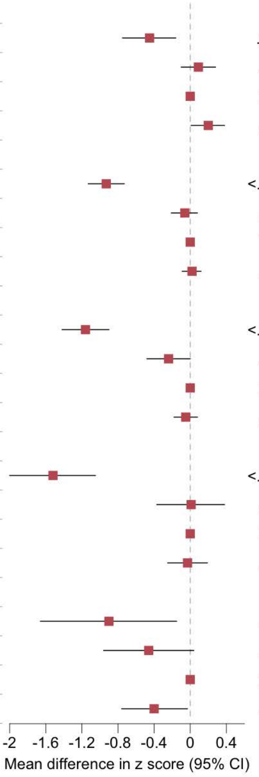

eFigure 2. The Association Between Surgery-to-Conception Interval and Pregnancy Weight Gain Among Pregnancies With a History of Gastric Bypass (N=5486)

[a]Z-score refers to the mean pregnancy weight gain z-score observed in the data, not predicted from the regression model.
[b]Corresponding weight gain z-score in kilograms at 40 weeks.
[c]Mean difference in z score estimated from the multivariable linear regression model with a robust sandwich estimator, adjusted for maternal age and parity.

© 2023 Xu H et al. *JAMA Network Open.*

Surgery-to-conception interval (years)	No of pregnancies	Mean pregnancy weight gain Z-score[a]	Kilogram[b]	Mean difference in z score (95% CI)[c]	P value
**Normal weight**					
<1	45	-0.58	11.7	-0.79 (-1.26, -0.31)	.001
1 to <2	69	0.08	14.6	-0.13 (-0.53, 0.26)	.50
2 to <4	53	0.21	15.2	Ref	NA
≥4	9	0.01	14.3	-0.19 (-0.93, 0.54)	.60
**Overweight**					
<1	108	-1.01	8.5	-1.03 (-1.37, -0.69)	<.001
1 to <2	102	-0.12	13.0	-0.11 (-0.42, 0.20)	.49
2 to <4	117	-0.02	13.6	Ref	NA
≥4	43	0.06	14.1	0.10 (-0.31, 0.51)	.62
**Obese class I**					
<1	62	-0.60	7.8	-0.58 (-1.00, -0.17)	.006
1 to <2	70	0.25	13.2	0.25 (-0.07, 0.57)	.12
2 to <4	75	-0.03	11.2	Ref	NA
≥4	27	-0.40	8.9	-0.40 (-0.95, 0.15)	.15
**Obese class II**					
<1	17	-0.85	4.2	-0.79 (-1.49, -0.09)	.03
1 to <2	20	0.45	13.0	0.46 (-0.04, 0.95)	.07
2 to <4	36	-0.04	9.2	Ref	NA
≥4	16	-0.03	9.3	-0.07 (-1.02, 0.88)	.88
**Obese class III**					
<1	5	-0.51	4.4	-0.42 (-1.35, 0.51)	.36
1 to <2	10	0.71	14.3	0.55 (-0.35, 1.45)	.23
2 to <4	12	-0.07	7.4	Ref	NA
≥4	9	-0.35	5.5	-0.29 (-1.10, 0.52)	.48

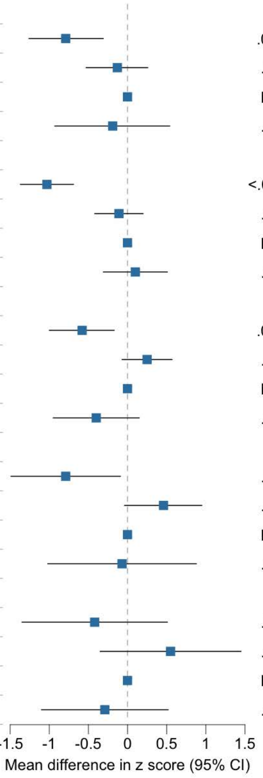

eFigure 3. The Association Between Surgery-to-Conception Interval and Pregnancy Weight Gain Among Pregnancies With a History of Sleeve Gastrectomy (N=905)

[a]Z-score refers to the mean of pregnancy weight gain z-score observed in the data, not predicted from the regression model.
[b]Corresponding weight gain z-score in kilograms at 40 weeks.
[c]Mean difference in *z* score was estimated from the multivariable linear regression model with a robust sandwich estimator, adjusted for maternal age and parity.

© 2023 Xu H et al. *JAMA Network Open.*

Surgery-to-conception interval (years)	No of pregnancies	Mean pregnancy weight gain Z-score[a]	Kilogram[b]	Mean difference in z score (95% CI)[c]	P value
**Normal weight**					
<1	62	-0.45	12.2	-0.48 (-0.85, -0.11)	.01
1 to <2	130	-0.06	13.9	-0.09 (-0.39, 0.21)	.54
2 to <4	157	0.02	14.3	Ref	NA
≥4	94	0.14	14.9	0.13 (-0.18, 0.44)	.41
**Overweight**					
<1	133	-1.01	8.5	-0.93 (-1.21, -0.66)	<.001
1 to <2	196	-0.09	13.2	0.00 (-0.22, 0.22)	.98
2 to <4	295	-0.11	13.1	Ref	NA
≥4	300	0.02	13.8	0.16 (-0.02, 0.34)	.08
**Obese class I**					
<1	91	-0.71	7.2	-0.90 (-1.25, -0.55)	<.001
1 to <2	87	0.05	11.8	-0.07 (-0.37, 0.24)	.67
2 to <4	180	0.14	12.4	Ref	NA
≥4	239	0.05	11.8	-0.05 (-0.27, 0.16)	.62
**Obese class II**					
<1	30	-1.34	1.9	-1.62 (-2.27, -0.97)	<.001
1 to <2	24	0.10	10.2	-0.14 (-0.74, 0.46)	.65
2 to <4	46	0.25	11.4	Ref	NA
≥4	106	0.00	9.5	-0.23 (-0.65, 0.20)	.29
**Obese class III**					
<1	7	-0.18	6.6	-0.39 (-1.39, 0.61)	.44
1 to <2	15	0.39	11.2	0.11 (-0.53, 0.76)	.73
2 to <4	16	0.29	10.3	Ref	NA
≥4	37	-0.17	6.7	-0.46 (-1.09, 0.17)	.15

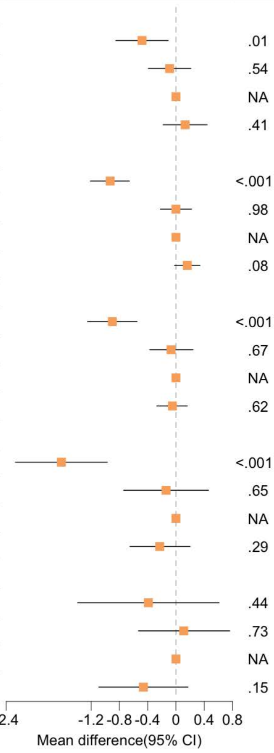

eFigure 4. The Association Between Surgery-to-Conception Interval and Pregnancy Weight Gain Among Nulliparous Women With a History of Bariatric Surgery (N=2245)

[a]Z-score refers to the mean pregnancy weight gain z-score exactly observed in the data, not predict from the regression model.
[b]Corresponding weight gain z-score in kilogram at 40 weeks.
[c]Mean difference in *z* score was estimated from the multivariable linear regression model with a robust sandwich estimator, adjusted for maternal age.

© 2023 Xu H et al. *JAMA Network Open.*

Surgery-to-conception interval (years)	No of pregnancies	Mean pregnancy weight gain: Z-score[a]	Mean pregnancy weight gain: Kilogram[b]	Mean difference in z score (95% CI)[c]	P value
**Normal weight**					
<1	92	-0.83	10.7	-0.46 (-0.80, -0.12)	.008
1 to <2	208	-0.17	13.4	0.19 (-0.03, 0.40)	.09
2 to <4	287	-0.36	12.6	Ref	NA
≥4	269	-0.22	13.2	0.14 (-0.07, 0.36)	.20
**Overweight**					
<1	195	-1.14	7.9	-0.90 (-1.11, -0.69)	<.001
1 to <2	259	-0.33	11.9	-0.09 (-0.25, 0.07)	.26
2 to <4	529	-0.24	12.4	Ref	NA
≥4	699	-0.31	12.0	-0.07 (-0.19, 0.05)	.26
**Obese class I**					
<1	103	-1.18	4.8	-1.07 (-1.37, -0.77)	<.001
1 to <2	107	-0.16	10.4	-0.05 (-0.29, 0.19)	.70
2 to <4	265	-0.11	10.7	Ref	NA
≥4	530	-0.20	10.1	-0.08 (-0.23, 0.06)	.26
**Obese class II**					
<1	38	-1.42	1.5	-1.21 (-1.75, -0.68)	<.001
1 to <2	39	0.11	10.3	0.31 (-0.02, 0.64)	.06
2 to <4	113	-0.20	8.1	Ref	NA
≥4	231	-0.15	8.4	0.04 (-0.19, 0.28)	.72
**Obese class III**					
<1	12	-0.95	2.0	-1.00 (-1.75, -0.25)	.009
1 to <2	11	-0.23	6.3	-0.29 (-0.92, 0.33)	.36
2 to <4	36	0.04	8.2	Ref	NA
≥4	123	-0.25	6.1	-0.31 (-0.70, 0.07)	.11

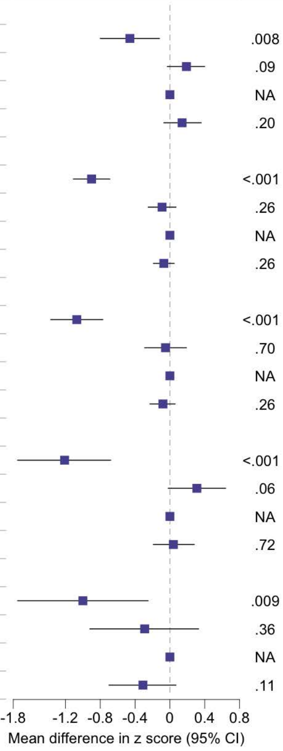

eFigure 5. The Association Between Surgery-to-Conception Interval and Pregnancy Weight Gain Among Parous Women With a History of Bariatric Surgery (N=4146)

[a]Z-score referred to the mean of pregnancy weight gain z-score exactly observed in the data, but not predict from the regression model.

[b]Corresponding weight gain z-score in kilogram at 40 weeks.

[c]Mean difference in *z* score was estimated from the multivariable linear regression model with robust sandwich estimators with adjustments for maternal age.

© 2023 Xu H et al. *JAMA Network Open.*

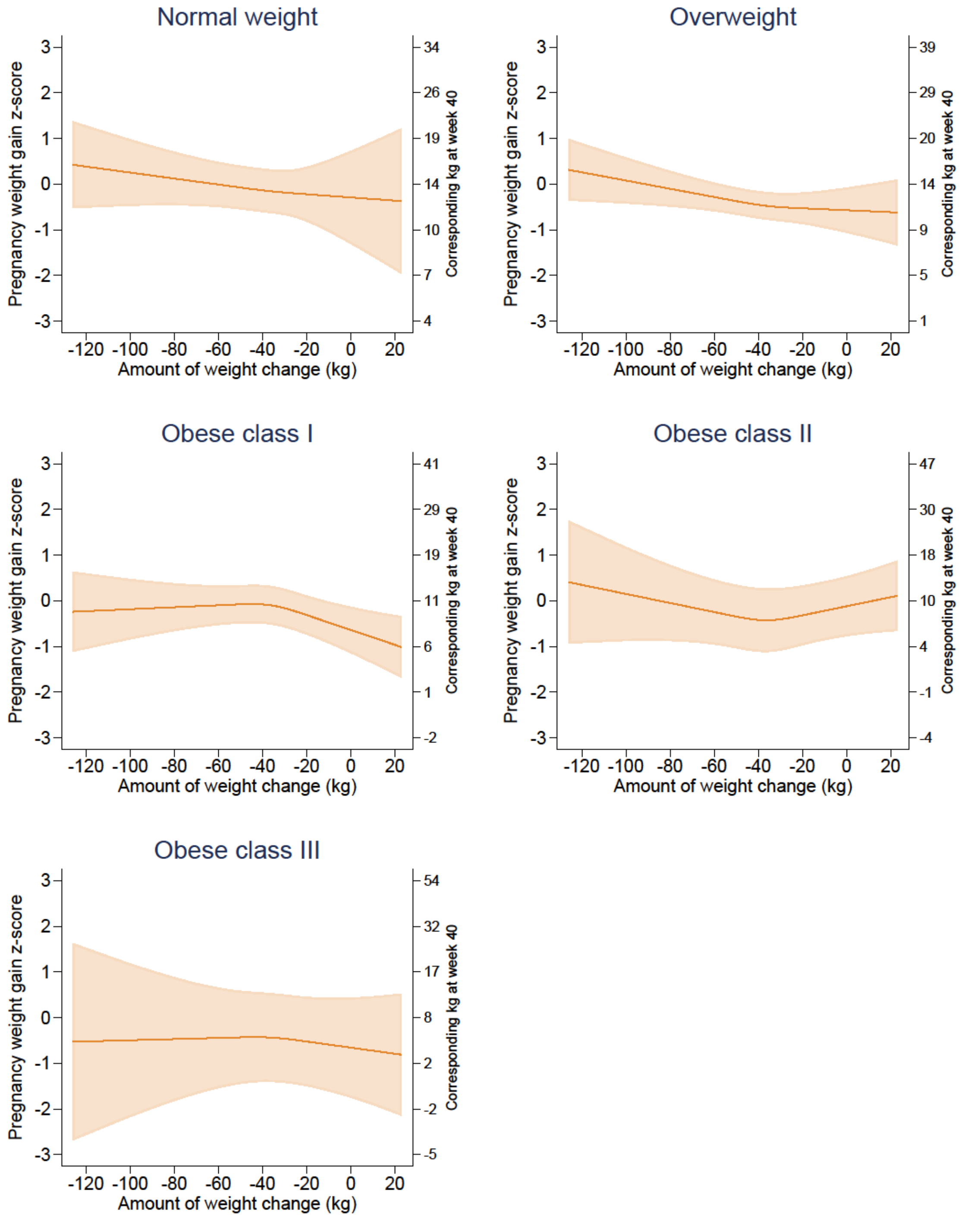

eFigure 6. Predicted Means of Pregnancy Weight Gain *z* Scores by Surgery-to-Conception Weight Change (N=6391)

Note: After excluding the underweight group, our analysis included 6391 pregnancies.
The solid lines represent the point estimates and the shadow represents the 95% confidence interval. Predicted means were set at the population average for maternal age and parity. The left y-axis shows pregnancy weight gain z-scores. The right y-axis represents the corresponding weight gain in kg at gestational week 40. Weight loss was modelled using a 3-knot restricted cubic spline.

© 2023 Xu H et al. *JAMA Network Open.*
