# Supplementary material for: Pregnancy Weight Gain After Gastric Bypass or Sleeve Gastrectomy
Source: JAMA Netw Open. 2023 Dec 5;6(12):e2346228. doi: 10.1001/jamanetworkopen.2023.46228 (PMC10698625; doi:10.1001/jamanetworkopen.2023.46228)
Supplement: Supplement 2. — Data Sharing Statement [file jamanetwopen-e2346228-s002.pdf]

Data Sharing Statement

Xu. Pregnancy Weight Gain After Gastric Bypass or Sleeve Gastrectomy. *JAMA Netw Open*. Published December 05, 2023. doi:10.1001/jamanetworkopen.2023.46228

Data
Data available: No
